# A year of monitoring 20 mesophilic full-scale bioreactors reveals the existence of stable but different core microbiomes in bio-waste and wastewater anaerobic digestion systems

**DOI:** 10.1186/s13068-018-1195-8

**Published:** 2018-07-19

**Authors:** Magdalena Calusinska, Xavier Goux, Marie Fossépré, Emilie E. L. Muller, Paul Wilmes, Philippe Delfosse

**Affiliations:** 1 Environmental Research and Innovation Department, Luxembourg Institute of Science and Technology, 41 rue du Brill, 4422 Belvaux, Luxembourg; 2 Eco-Systems Biology Group, Luxembourg Centre for Systems Biomedicine, University of Luxembourg, 7 avenue des Hauts-Fourneaux, 4362 Esch-sur-Alzette, Luxembourg; 3 Department of Microbiology, Genomics and the Environment, Université de Strasbourg, CNRS, GMGM, UMR 7156, Strasbourg, France

**Keywords:** Biogas, Microbial communities, Small rRNA gene amplicon high-throughput sequencing

## Abstract

**Background:**

Anaerobic digestion (AD) is a microbe-driven process of biomass decomposition to CH_4_ and CO_2_. In addition to renewable and cost-effective energy production, AD has emerged in the European Union as an environmentally friendly model of bio-waste valorisation and nutrient recycling. Nevertheless, due to the high diversity of uncharacterised microbes, a typical AD microbiome is still considered as “dark matter”.

**Results:**

Using the high-throughput sequencing of small rRNA gene, and a monthly monitoring of the physicochemical parameters for 20 different mesophilic full-scale bioreactors over 1 year, we generated a detailed view of AD microbial ecology towards a better understanding of factors that influence and shape these communities. By studying the broadly distributed OTUs present in over 80% of analysed samples, we identified putatively important core bacteria and archaea to the AD process that accounted for over 70% of the whole microbial community relative abundances. AD reactors localised at the wastewater treatment plants were shown to operate with distinct core microbiomes than the agricultural and bio-waste treating biogas units. We also showed that both the core microbiomes were composed of low (with average community abundance ≤ 1%) and highly abundant microbial populations; the vast majority of which remains yet uncharacterised, e.g. abundant candidate *Cloacimonetes*. Using non-metric multidimensional scaling, we observed microorganisms grouping into clusters that well reflected the origin of the samples, e.g. wastewater versus agricultural and bio-waste treating biogas units. The calculated diversity patterns differed markedly between the different community clusters, mainly due to the presence of highly diverse and dynamic transient species. Core microbial communities appeared relatively stable over the monitoring period.

**Conclusions:**

In this study, we characterised microbial communities in different AD systems that were monitored over a 1-year period. Evidences were shown to support the concept of a core community driving the AD process, whereas the vast majority of dominant microorganisms remain yet to be characterised.

**Electronic supplementary material:**

The online version of this article (10.1186/s13068-018-1195-8) contains supplementary material, which is available to authorized users.

## Background

Microbial resource management (MRM), which refers to an optimal management of microbes with the aim to develop new products and to improve existing bio-processes, is at basis of numerous strategies in different domains, including renewable energy production, nutrient and water recycling, environmental safety and health [1, 2]. Anaerobic digestion is an example of an efficient MRM application, which relies on different classes of fermentative and syntrophic bacteria’s interactions with methanogens to decompose organic compounds in an anaerobic environment into CO_2_ and CH_4_; the latter being an energy carrier [3]. With an estimated number of over 30,000 industrial installations worldwide, AD provides a permanent power production of 10,000 MW [4], and thus stable operation is highly desired. In addition to renewable and cost-effective energy production, AD has emerged in the European Union as an environmentally friendly model of bio-waste valorisation allowing both nutrient recovery from digestion residue, and a reduction in greenhouse gas emissions (in comparison to, e.g. aerobic composting or landfilling [5]).

In Luxembourg, the government has supported research and encouraged farmers and industries to produce energy from biomass through the AD process. More recently, the Third Industrial Revolution (TIR) strategy study by J. Rifkin mentions the important role that biogas can play in Luxembourg to complement the intermittent solar and wind power, especially through energy storage, on-demand energy production, or through the power-to-gas technology (http://www.troisiemerevolutionindustrielle.lu/). The same study encourages the circular bio-economy to be included in the food production systems, where biogas would be at the heart of a waste-as-nutrients circular process for a sustainable and circular agriculture. Anaerobic digestion has long been used to stabilize organic materials, mainly manure and sewage sludge, but nowadays its range of feeding substrate has been broadened to bio-waste (e.g. agricultural, municipal and food) and dedicated energy crops [6]. Whereas energy crops, including maize and immature cereals, are in most cases highly digestible substrates, competition may arise with their use as feed or food. On the contrary, according to the European Biogas Industry Association (EUBIA), estimated biogas yield (can vary according to the total solid content and biomass composition) from municipal solid waste (MSW) is similar to ley crops and can be twice higher than animal manure (http://www.eubia.org/cms/wiki-biomass/anaerobic-digestion/). Therefore, having seen the growing streams of organic waste as opportunity, in Luxembourg MSW is now converted into clean biogas which is directly injected into the local gas network. As an example, around 35,000 tonnes of organic waste corresponding to 44% of organic household waste are converted yearly into methane in Mondercange biogas plant in southern Luxembourg.

Anaerobic digestion is a microbe-driven process; however, typical AD reactor microbiome is still considered as “dark matter”, mainly due to the huge diversity of uncharacterised microbes representing in many cases candidate phyla [7]. Recently, through the use of 16S ribosomal RNA (rRNA) gene high-throughput amplicon sequencing and/or metagenomics, a number of studies have attempted to explore the microbial community ecology of AD, e.g. [3, 8–14]. Although these studies have provided great insights into the structure, dynamics and functionality of microbial communities, most of them were limited to the municipal/industrial wastewater installations or farm reactors where animal manure and/or energy crops were the main feeding substrates. MSW-supplemented AD reactors were given much less attention (e.g. [12]) most probably due to the lower number of waste-valorising installations. Linking the dynamics of AD microbial community structures (e.g. using microbial ecology parameters such as community richness, diversity and evenness) with the performance of the AD system has also yielded conflicting results [15–17]. On the one hand, higher microbial diversity is considered as a reservoir of microbes with redundant metabolic pathways, which is desirable to ensure a functional stability of microbial communities in case of the changing environment [18]. Thus, higher species diversity is often correlated with more stable AD reactors [17]. On the other hand, functional stability might also be conferred by less diverse communities possibly expressing complementary pathways (niche complementarity [19]). In this sense, by avoiding direct competition over the exploitation of available resources, lower species richness could sustain higher structural stability of such microbial communities. While, highly enriched communities might be more quickly destabilised by, e.g. viral predation [20], a stable core community composed of roughly 63 abundant genus-level operational taxonomic units (OTUs), accounting for 68% of relative community abundance, has recently been reported for activated sludge ecosystem [21]. Six-year monitoring survey of 32 Danish full-scale AD reactors also showed that out of the huge diversity of microbes merely 300 OTUs represented 80% of total rRNA reads across reactors [8]. While, the high overall diversity of microbes reported in different studies (e.g. in a range of 3000–5000 OTUs [8, 9, 11]) mitigates our efforts of their functional characterisation which is practical for only a limited fraction of species in a given system, the question to ask is whether the whole diversity really matters in anaerobic reactors? Can we select a core of microorganisms common to most AD systems (e.g. valorising agricultural by-products, energy crops, animal manure, sewage sludge and MSW, etc.) that would be structurally stable in different reactors? If yes, their further characterisation should become a research priority to show whether these core microbes reflect the population of putative key microbes to the AD process.

To address these questions, we first aimed to characterise the structures and stabilities of microbial communities, including bacteria and archaea, involved in biogas production in 20 full-scale AD bioreactors that were monitored over 1-year period at regular monthly time intervals. Second, using the core community concept, we intended to indicate putatively important organisms to the AD process. As a result, we confirmed that though being phylogenetically similar (represented by the same phyla) the different microbiomes were biogas unit-specific and structurally stable over the monitoring period. Furthermore, by associating the abundance and the distribution of microorganisms in the studied reactors we revealed the existence of a microbial core specific to the AD system. However, the microbial core of the full-scale bio-waste (agricultural and MSW) energy units largely differed from that of the AD reactors located at WWTPs. This confirms that these two main types of AD systems are operated by different microbial communities.

## Results and discussion

### Characteristics of the studied AD reactors

**Fig. 1:**
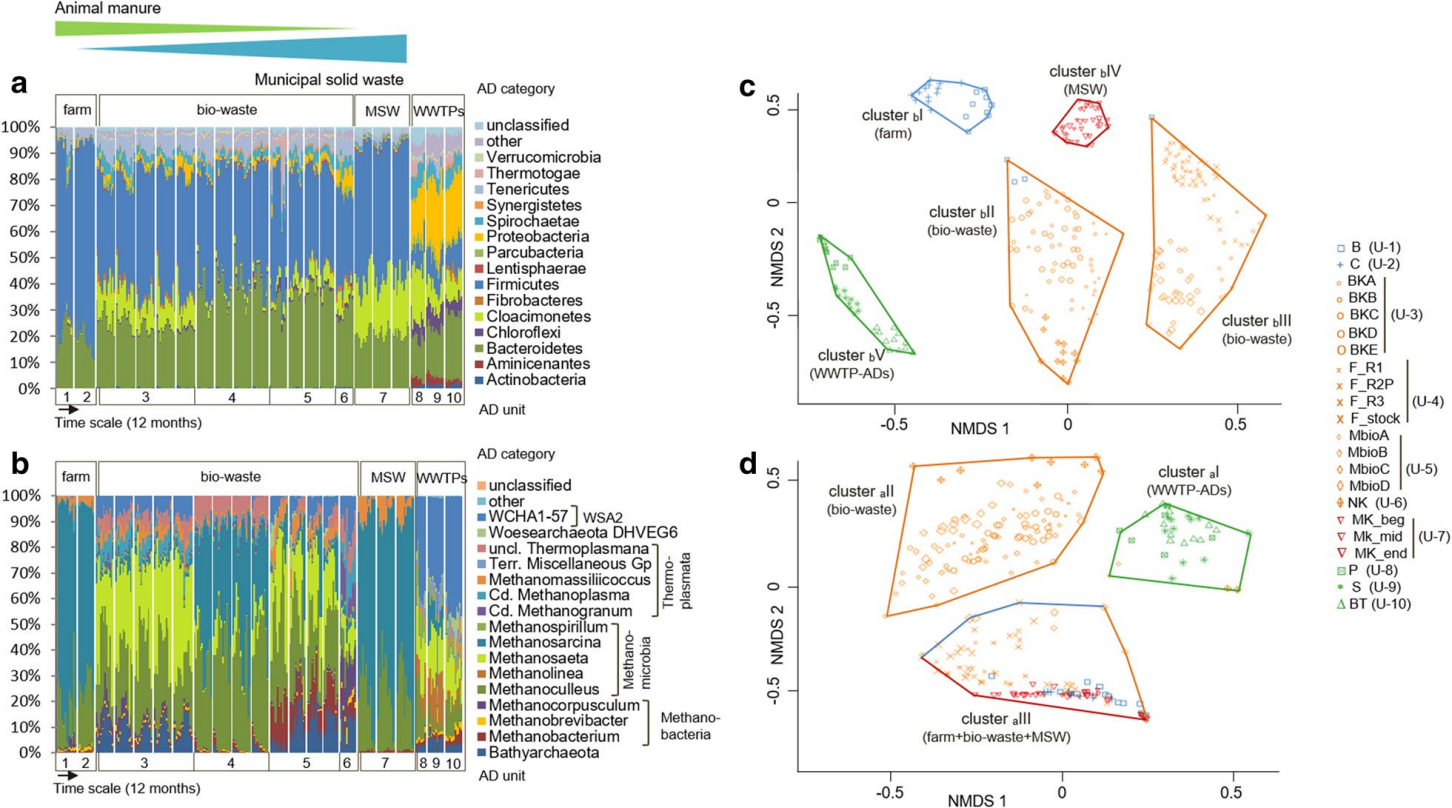
Taxonomic distribution and NMDS (Bray–Curtis dissimilarities in community structures) clustering analysis of **a**, **c** bacteria and **b**, **d** archaea for the 20 monitored mesophilic AD reactors (10 AD units) during the 1-year monitoring survey. For each reactor the 12 bars represent the monthly progress of the taxonomic distribution over the 1-year survey (**a**, **b**). In the case of the units 3, 4, 5, and 7 more than one reactor was analysed (see Table 1 for details). The AD categories refer to: “farm”—farm reactors fed mainly with agricultural residues, “bio-waste”—reactors treating bio-waste (a mixture of agricultural residues including manure and municipal and industrial bio-waste), “MSW”—a biogas unit treating sorted municipal solid waste and green waste and “WWTP-ADs”—anaerobic digesters of WWTPs (fed with sewage-activated sludge). An approximate ratio of animal manure to MSW in the reactor feed for the different reactors is shown above graph **a**. For NMDS graphs stress value and *R*^2^ for the two configurations equalled 0.2, 0.9 and ANOSIM R global was equal to 0.9 and 0.8, with *p* < 0.001, for bacteria and archaea, respectively

In total, 20 different full-scale mesophilic (temp. range from 33 to 44 °C) anaerobic reactors operating at 10 different biogas units (U-1–U-10) located in Belgium and Luxembourg were monitored during 1 year in regular monthly time series (Table 1). According to the main feeding substrates, the studied units were aggregated into four main AD categories, including: farm reactors fed mainly with agricultural residues (U-1 and U-2; acronym farm), reactors treating bio-waste (a mixture of agricultural residues including manure supplemented with MSW; U-3–U-6; bio-waste), a biogas unit treating uniquely sorted MSW including green waste (mostly garden and park residues; U-7; MSW), and anaerobic digesters of WWTPs (fed with sewage-activated sludge; U-8–U-10; WWTP-ADs). This classification scheme is stipulated and it aimed at reflecting the ordering based on the complexity/specificity of the feeding substrates (for the categories farm, bio-waste and MSW it reflects the decreasing ratio of manure towards MSW utilisation in the reactors; Fig. 1). All the reactors excluding U-7 (MSW) and WWTP-ADs were supplemented with different ratio of cattle manure (Table 1, Fig. 1). Reactor categories corresponding to farm, bio-waste and MSW will be commonly referred to as agricultural and bio-waste treating units, through the manuscript. Respectively, WWTP-ADs will often be discussed as a separate category. Except for U-7 which was operated as horizontal plug-flow-type (PFR), the other reactors were all operated as completely stirred tank reactors (CSTRs, Additional file 1: Figure S1). U-7 was also the only unit equipped with a separate hydrolysis box (partially covered, non-heated storage room where MSW was stored for 2–3 days before being fed to the reactor), and the anaerobic digestion process was a dry-type (solid content over 15%). In the case of units U-3, U-4 and U-5 several digesters and post-digesters located at the same biogas unit, as well as storage tanks were included in the analysis. For the horizontal rectangular PFR-type U-7, the inlet (MK-beg), outlet (MK-end) and the middle part (MK-mid) were monitored, showing no significant changes in species composition between the different parts (see below). During the time of the monitoring, all reactors were stably operated and none of them reported overt failures, including the MSW-supplemented reactor receiving the highly diversified feeding substrates (Table 1 and personal communication with plant managers). The sludge retention time was in a range of 2–3 weeks in the case of the WWTP-ADs and 5–12 weeks for the remaining reactors. A wide range in physicochemical parameters was observed between all the reactors with pH ranging between 7.1 (WWTP-ADs) and 8.0 (farm), total inorganic carbon (TIC) between 1.2 (WWTP-ADs) and 6.2 (bio-waste; m^3^ of CO_2_/m^3^ of slurry), ammonium–nitrogen (NH_4_–N) between 0.4 (WWTP-ADs) and 5.0 (bio-waste; kg NH_4_–N/m^3^ of sludge), total solids (TS) between 2.5 (WWTP-ADs) and 22.2 (MSW; % of TS/fresh sludge [w/w]), and volatile solids (VS) between 47.7 (WWTP-ADs) and 72.9 (farm; % of VS/% of total solid [w/w]). Details of these parameters values and volatile fatty acids (VFAs) concentrations at different sampling points are provided in Additional file 2: Table S1.

**Table 1:** Studied AD reactors and their physicochemical characteristics

Reactor type	Main feeding substrate	AD unit	Reactor acronym	Reactor category	pH	Temp. range °C	TIC	NH_4_–N	TS	VS
On-farm AD reactors fed mainly with agricultural residues	Maize and grass silages, cow manure and slurry (around 70%), bread	U-1	B	Fd	7.9 ± 0.2	39–40	3.4 ± 0.9	3.3 ± 0.9	8.6 ± 2.2	72.1 ± 4.8
	Maize silage, cow manure (around 70%), minor food waste	U-2	C	Fd	8.0 ± 0.1	42–44	4.7 ± 0.6	3.9 ± 1.1	10.2 ± 1.4	72.6 ± 2.9
AD reactors treating a mixture of agricultural residues (mainly manure) and bio-waste	Cow manure and slurry, grass, maize and cereal silages, diary waste	U-3	BKA	Fd-1	7.6 ± 0.2	33–39	2.7 ± 0.4	2.1 ± 0.6	8.3 ± 0.6	72.9 ± 2.9
			BKB	Fd-2	7.7 ± 0.2		2.9 ± 0.6	2.1 ± 0.6	8.0 ± 0.9	72.5 ± 2.9
			BKC	Pd-1	7.9 ± 0.2		3.3 ± 0.3	2.6 ± 0.9	7.2 ± 0.3	69.5 ± 1.9
			BKD	Pd-2	7.9 ± 0.2		3.3 ± 0.4	2.6 ± 0.7	7.3 ± 0.4	69.1 ± 1.8
			BKE	St	7.8 ± 0.2	nh	3.6 ± 0.3	2.9 ± 0.5	6.7 ± 0.5	67.1 ± 1.9
	Cow manure and slurry, cereal waste, waste from food industries, maize silage, grass	U-4	F-R1	Fd-1	7.8 ± 0.1	39	3.9 ± 0.5	3.5 ± 0.8	10.1 ± 0.8	65.9 ± 1.9
			F-R2P	Pd	7.9 ± 0.1		5.6 ± 0.5	4.5 ± 0.8	8.5 ± 0.6	61.2 ± 1.2
			F-R3	Fd-3	7.9 ± 0.1		4.8 ± 0.6	3.8 ± 0.8	8.8 ± 0.5	62.5 ± 1.6
			F-stock	St	7.9 ± 0.1	nh	6.2 ± 0.5	5.0 ± 0.9	7.6 ± 0.4	57.6 ± 2.5
	Manure, cereal waste, grass silage, organic waste from supermarkets and canteens	U-5	MbioA	Fd-1	7.5 ± 0.2	37–38	3.5 ± 0.7	3.6 ± 1.5	6.7 ± 0.9	66.5 ± 2.5
			MbioB	Fd-2	7.6 ± 0.2		2.3 ± 0.4	1.9 ± 1.1	10.8 ± 3.7	70.6 ± 2.6
			MbioC	Pd	7.6 ± 0.1		3.3 ± 0.5	2.7 ± 1.1	8.8 ± 0.9	68.1 ± 2.2
			MbioD	St	7.6 ± 0.2	nh	3.5 ± 0.4	2.8 ± 0.6	7.7 ± 1.7	65.7 ± 2.4
	Manure, green waste, maize silage, waste from food industries	U-6	NK	Fd	7.6 ± 0.3	36–37	3.4 ± 0.6	2.4 ± 0.8	7.8 ± 0.9	66.2 ± 2.2
Municipal and green waste treating AD reactors	Yard green waste (leaves, trees), organic waste from households and canteens	U-7	MK-beg	Fd	7.8 ± 0.2	39	4.9 ± 0.8	3.6 ± 1.5	21.6 ± 1.6	50.8 ± 2.7
			MK-mid		7.8 ± 0.2		4.6 ± 0.8	3.4 ± 1.5	22.2 ± 1.3	51.7 ± 3.2
			MK-end		7.8 ± 0.1		4.6 ± 0.9	3.5 ± 1.6	21.8 ± 1.7	50.7 ± 2.8
WWTP-specific AD reactors	Thickened sewage sludge from wastewater treatment	U-8	P	Fd	7.2 ± 0.1	35	1.3 ± 0.3	0.4 ± 0.5	2.5 ± 0.4	47.7 ± 3.3
		U-9	S	Fd	7.1 ± 0.1	36	1.2 ± 0.2	0.7 ± 0.5	3.8 ± 0.4	55.6 ± 1.8
		U-10	BT	Fd	7.2 ± 0.1	36	1.4 ± 0.2	1.3 ± 0.9	3.0 ± 0.2	49.0 ± 1.6

*Fd* fed digester, *Pd* post-digester, *St* storage tank, *TIC* total inorganic carbon (m^3^ of CO_2_/m^3^ of sludge), *NH*_*4*_*–N* total ammonium–nitrogen (kg NH_4_–N/m^3^ of sludge), *TS* total solids (% of total solid/fresh sludge [w/w]), *VS* volatile solids (% of volatile solid/ % of total solid [w/w], *nh* non-heated

### Taxonomic distribution of bacteria and archaea in AD reactors based on the 16S rRNA gene amplicons

In our study, a year of monitoring microbial communities (using a newly optimised protocols for bacterial and archaeal 16S rRNA gene amplicon sequencing; Additional files 3 and 4) revealed the existence of different but structurally stable microbiomes in wastewater and agricultural and bio-waste anaerobic digestion systems. Sequencing of bacterial 16S rRNA gene amplicons from over 250 samples (subsampled to 11,550 quality-trimmed reads per sample), resulted in a total of 5938 bacterial OTUs (_b_OTUs defined at 97% sequence similarity and represented by more than one sequence), representing 55 known and candidate phyla (Additional file 5: Table S5). Rarefaction curves observed based on the species richness did not reach the plateau; however, the rank-abundance curves showed that OTUs representing at least 0.008% of the bacterial community were detected (Additional file 6: Figure S6). In line with our results, previous studies of full-scale AD reactors reported a total number of bacterial OTUs to be in a range of 3000–5000 [9, 11]. Twelve bacterial phyla were represented in all analysed samples, and nine were at ≥ 1% average 16S rRNA gene amplicons reads abundance (decreasing order: *Firmicutes*, *Bacteroidetes*, *Cloacimonetes* previously known as “candidate phylum WWE1”, *Proteobacteria*, *Tenericutes*, *Spirochaetes*, *Aminicenantes*, *Chloroflexi*, *Parcubacteria*). Bacterial communities of agricultural and bio-waste treating units were mainly dominated by *Firmicute*s (46.4% ± 11.8 of sequencing reads per sample) and *Bacteroidetes* (28.5% ± 8.5) with a minor proportion of reads assigned to *Cloacimonetes* (8.0% ± 7.5) and *Tenericutes* (5.3% ± 3.0). WWTP-ADs had distinct bacterial communities with *Proteobacteria* (22.6% ± 4.3), *Bacteroidetes* (20.3% ± 4.5), *Cloacimonetes* (12.0% ± 6.2) and *Firmicute*s (9.5% ± 2.1; Fig. 1a) as principal phyla. While, in general, a more dominant phylum was represented by a higher number of _b_OTUs, some phyla like *Cloacimonetes* and *Proteobacteria* did not follow this trend. Only 18.7% of _b_OTUs were common to the two anaerobic digestion systems, while 53.7 and 27.6% of bacterial OTUs were specific to agricultural and bio-waste treating units and WWTP-ADs, respectively. The predominance of *Firmicutes* and *Bacteroidetes* has been previously shown (using both 16S rRNA amplicon sequencing and metagenomics) in many full-scale (mainly manure supplemented) AD reactors [3, 9, 13, 22]; while other taxonomic divisions including *Proteobacteria* and *Spirochaetes* [11] or *Chloroflexi* and *Proteobacteria* [23] dominated mainly WWTP-specific (sewage sludge-supplemented) AD reactors previously studied. The presence of candidate phylum *Cloacimonetes* has less often been reported in the literature, even though it clearly dominated bacterial communities in some full-scale digesters [24–26] and lab-scale reactors [27, 28].

Concerning archaea, the high-throughput analysis of quality-trimmed reads subsampled to 2070 reads per sample resulted in 89 archaeal OTUs (_a_OTU), out of which 66 were assigned to *Euryarchaeota* (86.6% of total reads), six to *Woesearchaeota* (0.6%), five to *Bathyarchaeota* (4.8%) and three to WSA2 (7.9%; Fig. 1b). The rarefaction curves based on the species richness reached the plateau, suggesting that the description of the archaeal diversity was nearly exhaustive (Additional file 6: Figure S6). *Methanomicrobia* (71.2% ± 19.2) and *Thermoplasmata* (11.0% ± 7.3; in U-6 they constituted 31.6% ± 5.8 of archaeal community) were prevailing classes in all AD units, while *Methanobacteria* accounted for around 4.3% ± 4.6 of all archaea. The highest abundance of *Bathyarchaeota* was detected in the ADs located at U-3 (8.3% ± 4.7) and U-5 (10.0% ± 6.2). Next to *Methanomicrobia* (44.2% ± 13.8), WCHA1-57 (39.0% ± 10.3) dominated in WWTP-ADs. In contrast to bacteria, around 56.2% of archaeal OTUs were common to the two main anaerobic digestion systems. In total 31.5% of _a_OTUs were specific to agricultural and bio-waste treating units and only 12.3% to WWTP-ADs.

### Diversity versus stability of bacterial populations in AD reactors

**Fig. 2:**
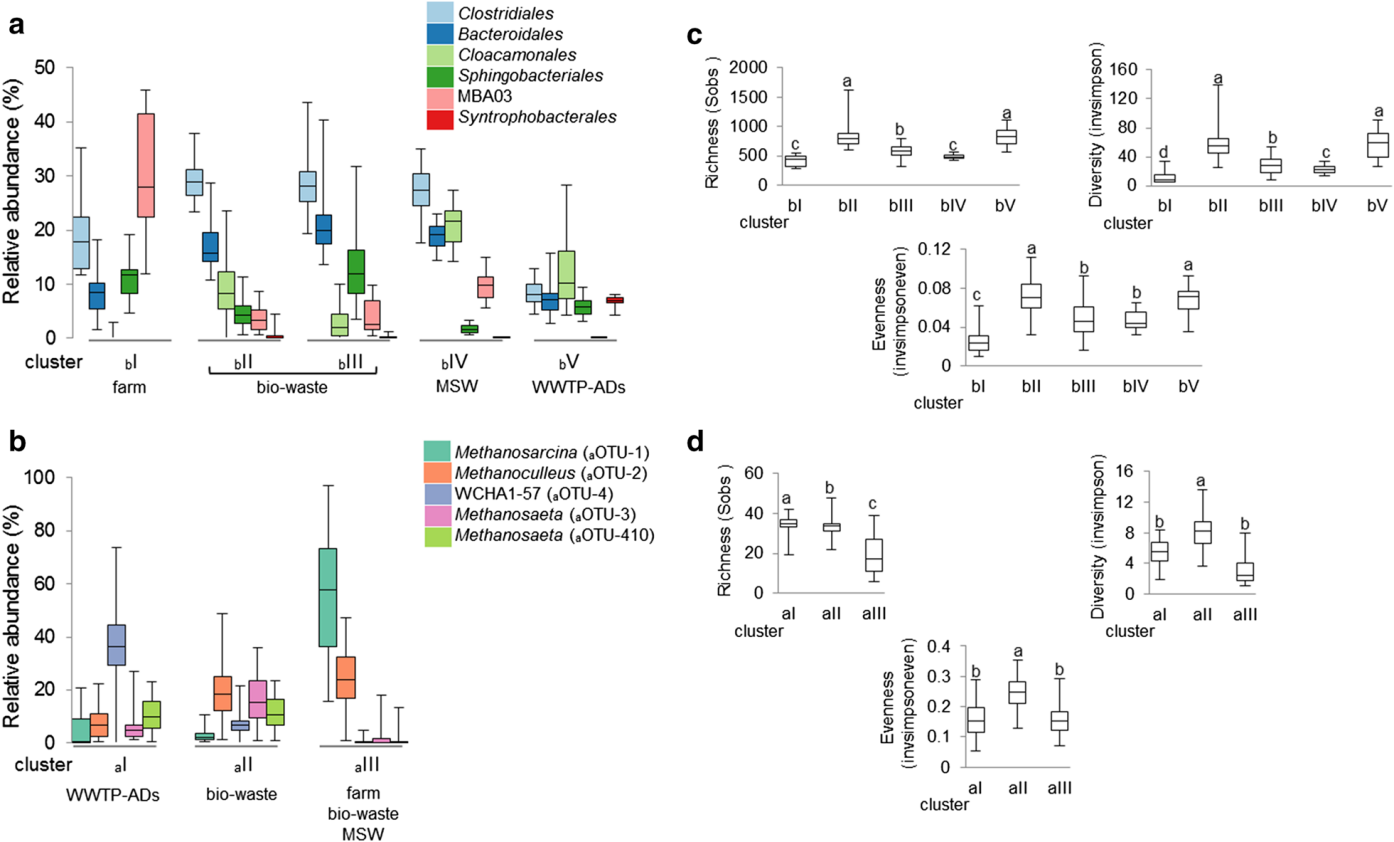
Representation of dominant microbial groups per cluster (**a**, **b**) and the diversity indices for microbial communities (**c**, **d**) analysed for the 20 monitored mesophilic AD reactors. The relative abundance of the dominant bacteria was shown at the order level (**a**) and archaea at the OTU level (**b**) and for each of the clusters. In the case of the richness (sobs index) and the diversity (invsimpson index), increased values indicate higher species richness and diversity, respectively, and correspond to the bacterial (**c**) and archaeal (**d**) clusters. For the evenness (invsimpsoneven index), values range from 0 (non-even) to 1 (even). The boxes represent the interquartile range and the error bars show the 95% confidence intervals; cluster _a_I (*n* = 33), _a_II (*n* = 105), _a_III (*n* = 113), _b_I (*n* = 23), _b_II (*n* = 80), _b_III (*n* = 89), _b_IV (*n* = 36), _b_V (*n* = 34). Statistical analyses were performed using Kruskal–Wallis test; boxplots holding the same label within a single panel do not differ significantly (*p* ≤ 0.05)

To analyse the variation in microbial communities within a single reactor and across different biogas units we used the non-metric multidimensional scaling (NMDS) of the calculated Bray–Curtis dissimilarities in community structures at the OTU level (Fig. 1c). The NMDS clustering of bacterial communities visibly separated (ANOSIM R global equalled 0.9 with *p* < 0.001) AD reactors treating activated sludge located at the WWTPs (cluster _b_V) from agricultural and bio-waste treating units (clusters _b_I to _b_IV), confirming the presence of different microbiomes in the two AD systems [13]. Additionally, clustering of samples resulted in biogas unit- and sampling time-specific segregation indicating the strong influence of operational conditions and confirming previous observations [3, 9, 10, 13]. The formation of the tight collections of points indicated that the variation over time of microbial communities within a reactor and/or AD plant is lower than in the cross-section. Former clustering analysis of 38 samples collected from 29 different full-scale AD installations indicated the dominance of either *Bacteroidales* or *Clostridiales* in two different clusters grouping mesophilic sludge digesters [9]. In our study, next to mainly *Clostridiales* and *Bacteroidales*-dominated clusters _b_II and _b_III (Fig. 2a), we show the presence of MBA03-dominated (Firmicutes) cluster _b_I specific to farm reactors, cluster _b_IV (MSW) where three bacterial orders including *Clostridiales*, *Cloacamonales* and *Bacteroidales* were co-abundant, and WWTP AD-specific cluster _b_V dominated by *Cloacamonales*, *Clostridiales*, *Bacteroidales*, *Sphingobacteriales* and *Syntrophobacterales*. The co-occurrence of MBA03 which was evidenced to be an electroactive genus [29] and *Methanosarcina* (see below), might suggest the direct interspecies electron transfer (DIET) to potentially be an effective form of syntrophy in farm methanogenic reactors [30].

**Fig. 3:**
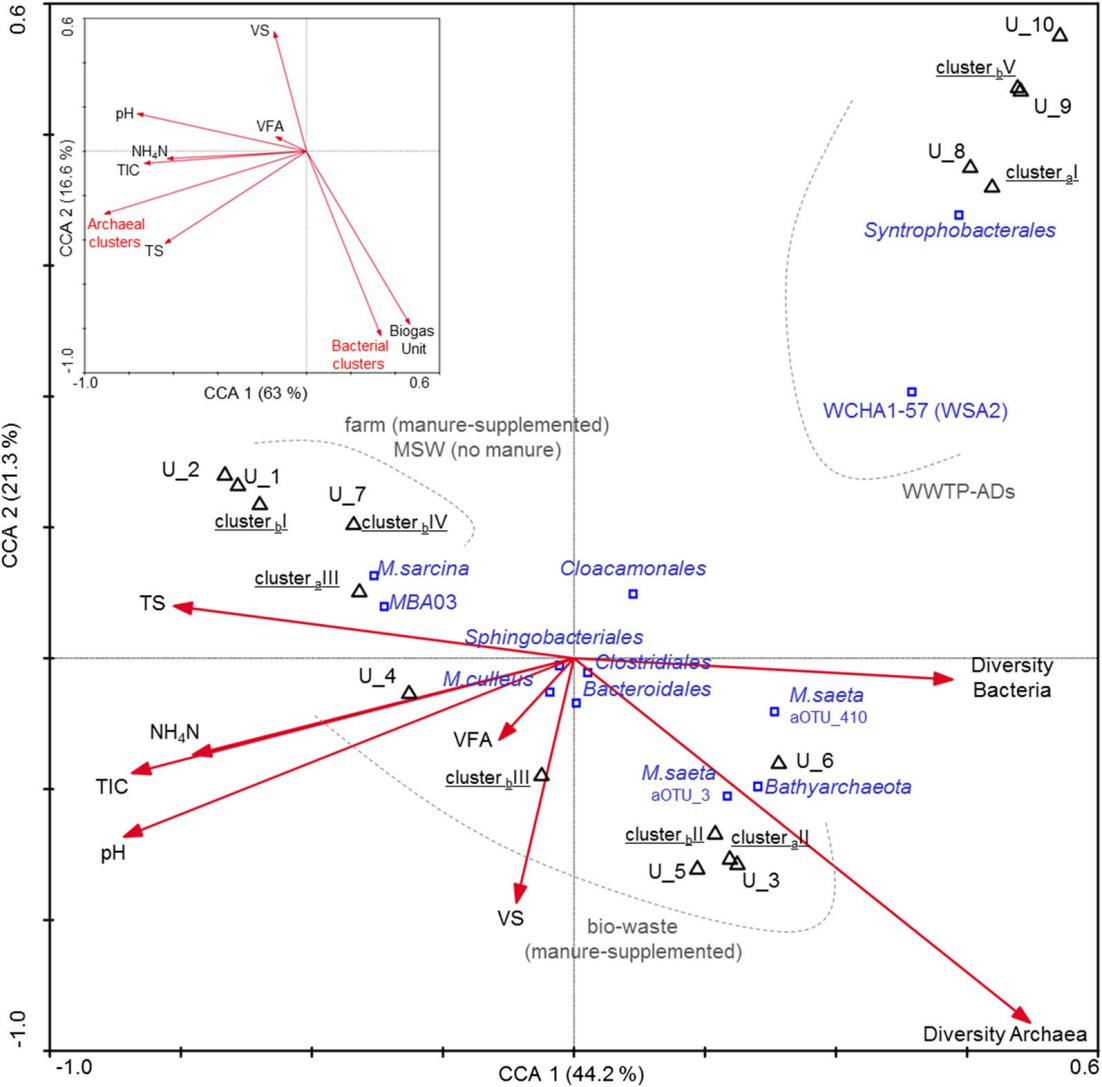
Canonical correspondence analysis (CCA) ordination diplot for bacterial and archaeal community clusters. Red vectors represent the influence of the process parameters (pH, total solids [TS], volatile solids [VS], total inorganic carbon [TIC], ammonium–nitrogen [NH_4_–N], total volatile fatty acids [VFAs]) and bacterial and archaeal diversity and richness indicators. Bold black triangles represent biogas units; small blue squares show some of the most abundant bacterial orders, archaeal genera or OTUs discussed in the manuscript. The pop up CCA in the left corner shows the influence of the process parameters on the archaeal and bacterial clustering

In terms of bacterial diversity, ADs reactors specific to WWTPs (cluster _b_V) were similar to the bio-waste units grouping in cluster _b_II (Fig. 2c; Additional file 6: Figure S7). Bacterial communities from farm reactors (cluster _b_I) and MSW (cluster _b_IV) were the least diverse and evenly distributed (*p* ≤ 0.05), even though U-7 was receiving highly diversified over 1-year MSW feed. In these reactors, bacterial diversity was significantly negatively correlated to mainly TS and NH_4_–N, TIC, total VFAs (mainly acetate) and pH (*p* ≤ 0.01; Fig. 3; Additional file 7: Table S6). While it has been shown that the initial community evenness favours community functionality under selective stress [18], communities dominated by single species could assure similar resistance to the perturbation, provided that they are tolerant to it. Both strategies were observed in the studied reactors: (1) less diverse communities that were generally dominated by single OTUs (clusters _b_I and _b_IV; interestingly they were correlated with higher TS), and (2) the more diverse that were characterised by a higher OTUs evenness (clusters _b_II and _b_III and _b_V). Interestingly, the stabilities of these communities were similar (Additional file 6: Figure S8), assuring stable operation (i.e. 7.0 ≤ pH ≤ 8.0, low VFAs concentrations and stable biogas production as reported by all plants operators) of the studied AD reactors during the monitoring period (Table 1 and Additional file 2: Table S1). Therefore, to what extent increased species diversity increases the functional stability of the AD system? What are the mechanisms that optimise diversity within a given biological community? According to the insurance hypothesis proposed by Yachi and Loreau [31], species richness at which specific ecosystem becomes functionally redundant largely depends on the way the different species interact and respond to the changing environment. Moreover, according to [32], the more a species is functionally dependent on the activity of another, fewer species are necessary to maintain ecosystem stability. In this sense, even highly productive communities might exhibit reduced species diversity [19], since the high functional redundancy required in more variable environments (e.g. soil, ocean) would no longer be essential in a relatively stable and highly specialised methanogenic world. These observations may suggest that a more diverse community does not necessarily mean a better adapted to anaerobic digestion. Indeed, U-7 receiving MSW was characterised with the highest community structural stability (Additional file 6: Figure S8A), that was at the same time one of the least diverse and even (in this case the dominant _b_OTU represented the candidate phylum *Cloacimonetes*).

### Archaeal community clusters and diversity patterns

Archaeal communities of WWTPs (cluster _a_I) differed significantly (ANOSIM R global equalled 0.9 with *p* < 0.001) from agricultural and bio-waste treating units (clusters _a_II and _a_III) and the separation was mainly attributed to the different NH_4_–N and TIC parameters (Figs. 1d and 3). The diversity patterns varied between the different AD reactors (Fig. 2d; Additional file 6: Figure S9); however, similar trends of diversity and richness were observed for archaeal communities from reactors characterised by the same type of a feeding regime. In general, archaeal cluster _a_II (bio-waste) was characterised by the highest species diversity and evenness in contrast to the cluster _a_III (mainly regrouping farm and MSW) that was lower (Fig. 2d). Interestingly, an increased relative abundance of *Bathyarchaeota* (_a_OTU-6) was positively correlated (*p* < 0.001) with increased archaeal diversity in the studied reactors (Fig. 3; Additional file 7: Table S6).

On average *Methanosarcina* was the most abundant and omnipresent archaeon (Figs. 1b and 2b); however, it dominated over *Methanosaeta* only in 46.8% of analysed samples (mainly cluster _a_III). As previously reported, its dominance was correlated with a higher concentration of acetate and ammonium–nitrogen (Additional file 7: Table S6; [12]), and a higher TS content of a reactor (Fig. 3). In the other half of the samples, two *Methanosaeta*_a_OTU-3 and _a_OTU-410 were co-abundant (mainly cluster _a_II and to a lower extent _a_I), and correlated with the increased abundance of *Bathyarchaeota*. Co-occurrence of *Bathyarchaeota* with some *Methanomicrobia*, including *Methanosaeta* has been previously reported across different terrestrial settings [33]. Miscellaneous *Crenarchaeota* Group (MCG) renamed to *Bathyarchaeota* has recently been shown to encode in its genome genes necessary for hydrogenotrophic and methylotrophic methanogenesis, including the methyl Co-A reductase [34]. Its co-dominance with *Methanosaeta* would thus assure the presence of the gene pool assuring the methane production by all three known methanogenic pathways, including acetoclastic, hydrogenotrophic and methylotrophic. The dominance of the unknown WSA2 archaeon (WCHA1-57, _a_OTU-4) in all studied WWTP-ADs (cluster _a_I) opposes to the study of 32 Danish full-scale reactors located at 20 WWTPs, where between 60 and 80% of reads were assigned to *Methanosaeta* [35]. Our result is unlikely to be an artefact resulting from the assay design, since this archaeon was not dominating elsewhere in the studied reactors. Moreover, the most represented archaeal group in the study of Rivière et al. [36] was affiliated with WSA2 as well.

### Distribution and stability of bacterial _b_OTUs across the studied full-scale AD reactors

**Fig. 4:**
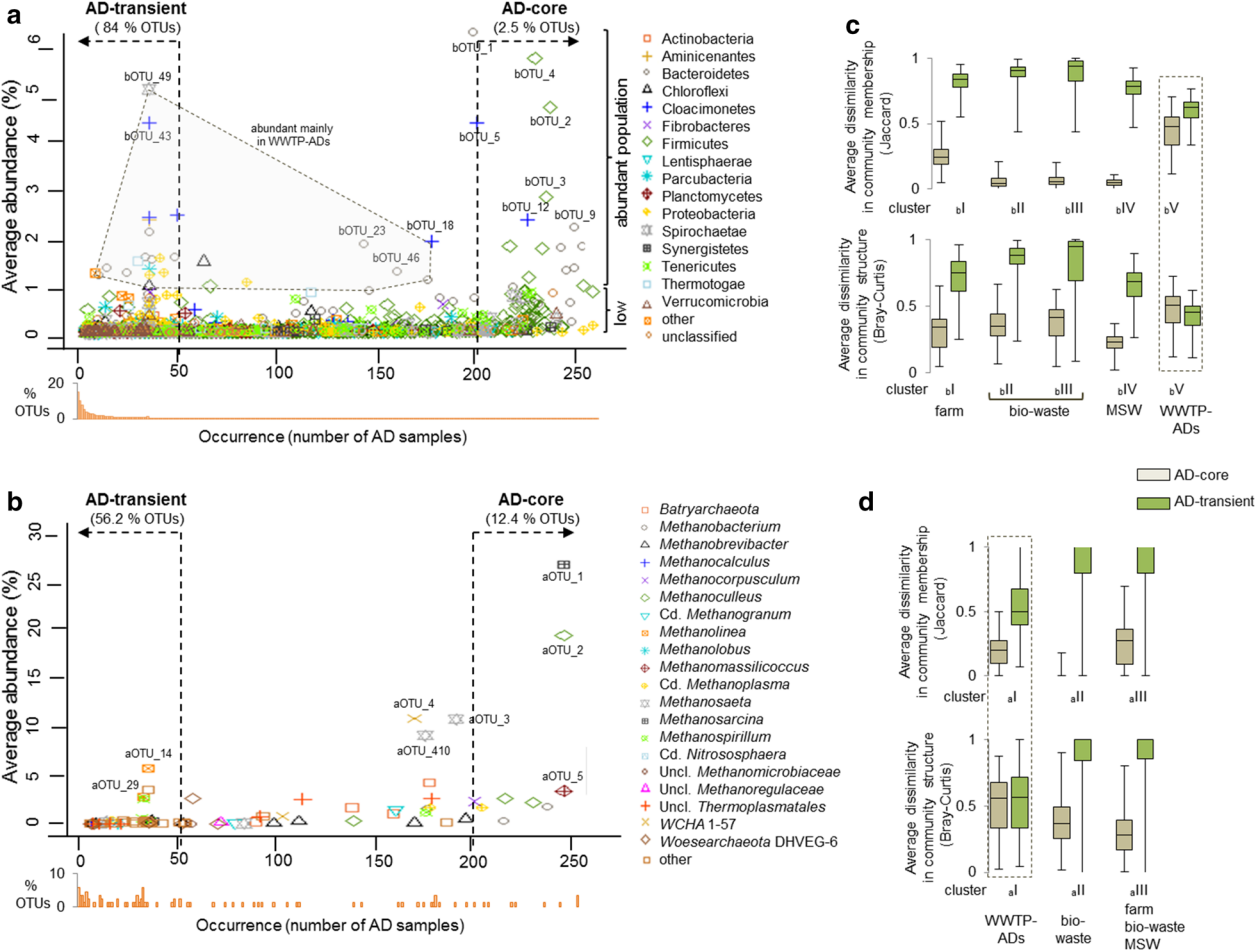
Average abundance versus occurrence plots (general AD-core and AD-transient microbiomes) for **a** bacterial and **b** archaeal OTUs. AD-core OTUs were defined as appearing in over 80% of the analysed sludge samples; AD-transient OTUs were defined as being present in less than 20% of the analysed samples. Density plots (orange bars) displayed below the graphs **a** and **b** represent the percentage of OTUs occurring in a specified number of samples. Community structural stability (Bray–Curtis dissimilarity) and membership (Jaccard dissimilarity) for AD-core and AD-transient populations of OTUs for **c** bacteria and **d** archaea (represented for bacterial and archaeal clusters). Values range from 0 (identical) to 1 (100% different). The boxes represent the interquartile range and the error bars show the 95% confidence intervals; cluster _a_I (*n* = 33), _a_II (*n* = 105), _a_III (*n* = 113), _b_I (*n* = 23), _b_II (*n* = 80), _b_III (*n* = 89), _b_IV (*n* = 36), _b_V (*n* = 34). Except for “WWTP-ADs” differences between the core and transient communities for the calculated indices were significant (Wilcoxon signed rank test with continuity; *p* < 0.05)

It has been proposed, that commonly occurring organisms appearing in most of the microbial communities associated with a particular environment, are likely important to the functioning of the whole community [37]. Revealing these stable and consistent components across different anaerobic digestion systems and defining the core (structural and functional) microbiome is important to the understanding of the whole process and could guide future manipulation of communities to attain a desired outcome. Detailed analysis of the clusters revealed that some OTUs were shared between all the reactors (except for the WWTP ADs), regardless the feeding regime (i.e. the ratio of manure to MSW in the feed). In line with [1], we initially defined as a general AD-core microorganisms occurring in 80% of the studied AD samples. Both abundant and rare (≤ 1% average reads abundance) OTUs were considered, and not only the top abundant per sample as previously proposed [38]. Except for WWTP-ADs that were shown to operate with a distinct core microbiome (see below), 2.5% of bacterial OTUs assigned to AD-core accounted for 70.3% ± 12.5 average bacterial read abundance in the reactors. AD-core _b_OTUs were typical AD members, mainly assigned to *Firmicutes* (82 OTUs) and *Bacteroidetes* (28 OTUs). As a general trend we confirmed that most of the dominant (with the highest average abundances across samples) bacterial OTUs were at the same time the most broadly distributed (Fig. 4a; [38]). However, there was also a considerable population of low abundant bacteria having the core community characteristics.

In line with the Pareto concept of species distribution in relation to their ecological tasks [39] applied in different microbial ecology studies [40, 41], abundant microorganisms (up to 20% top abundant species) would process 80% of the energy flux in the environment. In this sense, abundant AD-core microbes could be regarded as “key players” of the process, presumably performing the most important environmental tasks in their niche [1]. Indeed, an optimal microbial metabolism operating close to the Pareto value has recently been proposed for AD [42]. Nevertheless, the dominance in the community might not correlate with the activity of a specific microbe, meaning that bacterial abundances not always reflect growth rates or activity [43]. In that study, some of the low abundant bacteria seemed the most active in the community. Accordingly, low abundant AD-core populations of microbes might exert a disproportionately large effect on the functionality of the community by, e.g. performing a very specific task. Consequently, their persistence might be occasionally favoured; however, at a frequency high enough to retain their community membership [17]. As an example, syntrophic acetate-oxidizing bacteria (SAOB) have been shown to occupy a unique functional niche in the microbiome of some ADs (mainly operated at high NH_4_–N load), at the same time representing a low abundant community of microbes [44].

Consistent with previous reports [38], 84.0% of all bacterial OTUs were assigned to AD-transient _b_OTUs (defined as being present in less than 20% of analysed samples and mostly detected at single measurement points), representing 3.5% ± 3.8 of the relative bacterial abundances. They belonged mainly to *Firmicutes* (1742 OTUs), *Proteobacteria* (802 OTUs) and *Bacteroidetes* (615 OTUs). The remaining 790 bacterial OTUs that were neither classified as core nor transient were only shared between some bioreactors, and on average constituted 26.2% ± 10.0 of bacterial abundances in the different reactors. Roughly 150 of these _b_OTUs were common to all the bacterial clusters, and most of them did not persist in the reactor over time. A few of them were locally abundant (also in WWTP-ADs-core), including _b_OTU-18 classified to *Cloacimonetes* and several other *Bacteroidetes*_b_OTUs, e.g. _b_OTU-23 or _b_OTU-46.

To evaluate the extent to which the AD-core and AD-transient bacteria contribute to the observed bacterial diversity patterns and community structural stability in the studied AD reactors, we calculated separately for the core and transient populations the dissimilarity in community membership and structure over time. As a result, AD-core bacterial communities appeared quite stable in the studied reactors over the year, both in terms of the community membership and structure (except for WWTP-ADS; Fig. 4c; Additional file 6: Figure S10). By contrast, concerning the AD-transient communities, the dissimilarities in the community membership and structure were very high for all studied reactors. Except for WWTP-ADs, only 16 out of 4997 AD-transient _b_OTUs were abundant (≥ 1% of relative abundance) in at least one sample. Out of these, nine appeared at the same time point in serial reactors located at the same biogas unit, suggesting that they were supplied to the reactor with the influent streams. Only two of these transient _b_OTUS were detected in more than two consecutive samplings, suggesting that most of the AD-transient microbes were not able to develop on a longer term (at least not over 1 month period) in AD environment, and contrasting with previous findings revealed for WWTP ADs [38]. The observed differences might result from the sampling intervals that equalled 1 month (during 1 year) in our study versus 2–7 days (during 1 month) [38]. Therefore, while our core concept relates to the microbes that were present at least during 1 year in a reactor, what was observed in the other study can only be valid at monthly basis.

As stated by [1] and the Hubbell theory, plenty of rare species happen to be there, sometimes without much relation to the functionality of a particular environment. Therefore, even if we cannot exclude some of the transient microbes being important to the process, at this stage of our knowledge, we should probably first focus on characterising the most abundant and/or widespread core species.

### Distribution and stability of archaeal _a_OTUs across the studied full-scale AD reactors

AD-core archaea representing 12.4% of _a_OTUs (assigned exclusively to *Euryarchaeota*) accounted for 75.3% ± 19.4 of the relative archaeal reads abundances across samples (Fig. 4b). The presence of three _a_OTUs in all analysed reactors (at every sampling point) corresponding to *Methanosarcina* (_a_OTU-1), *Methanoculleus* (_a_OTU-2) and *Methanomassiliicoccus* (_a_OTU-5) assures the potential to produce methane through any of the three methanogenic pathways [45]. Out of the 50 _a_OTUs assigned as AD-transient archaea, some were locally abundant only in WWTP-ADs, confirming that this type of reactors operates with different archaeal core communities. They only accounted for an average of 0.4% ± 0.3 of the relative archaeal abundance across the agricultural and bio-waste treating units. The abundance of _a_OTUs that was neither assigned to AD-core nor AD-transient populations varied from 83.5% in the case of some WWTP-ADs, to as little as 0.1% for unit U-7 (MSW). For agricultural and bio-waste treating units these OTUs accounted for an average of 0.4% ± 0.3. This observation shows that in contrast to bacteria, mainly process-specific archaea are present in AD reactors.

Based on the calculated Jaccard and Bray–Curtis indices, core archaeal communities were less stable than bacterial cores (Fig. 4c, d). However, the higher standard variations mostly resulted from the lower species diversity and sparse occurrence of some archaea in the different reactors.

### Bacterial and archaeal core communities specific to WWTP-ADs

Until now, most attempts to characterise core bacterial communities of anaerobic reactors were mainly restricted to WWTP AD facilities. The very first trial was limited by the low throughput of Sanger sequencing [36]. According to that report, bacterial core was composed of six _b_OTUs affiliated with *Chloroflexi*, *Betaproteobacteria*, *Bacteroidetes* and *Synergistetes*. More recently, using a high-throughput 16S rRNA gene amplicon sequencing of 40 sludge samples collected from seven WWTP-ADs located in China, a total of 31 _b_OTUs (mainly assigned to *Bacteroidetes*, *Proteobacteria* and *Firmicutes*) with abundance greater than 3% of total reads in each digester were assigned to the core community [46]. Similarly to our results, by analysing 32 ADs located at 20 WWTPs in Denmark, Kirkegaard and collaborators [8] identified 300 abundant core organisms that accounted for 80% of reads. In that study, a few candidate phyla, including *Fermentibacteria* (Hyd-24-12), *Aminicenantes* (OP8) and *Atribacteria* (OP9) were present along with the commonly described *Firmicutes*, *Proteobacteria* and *Bacteroidetes*.

**Fig. 5:**
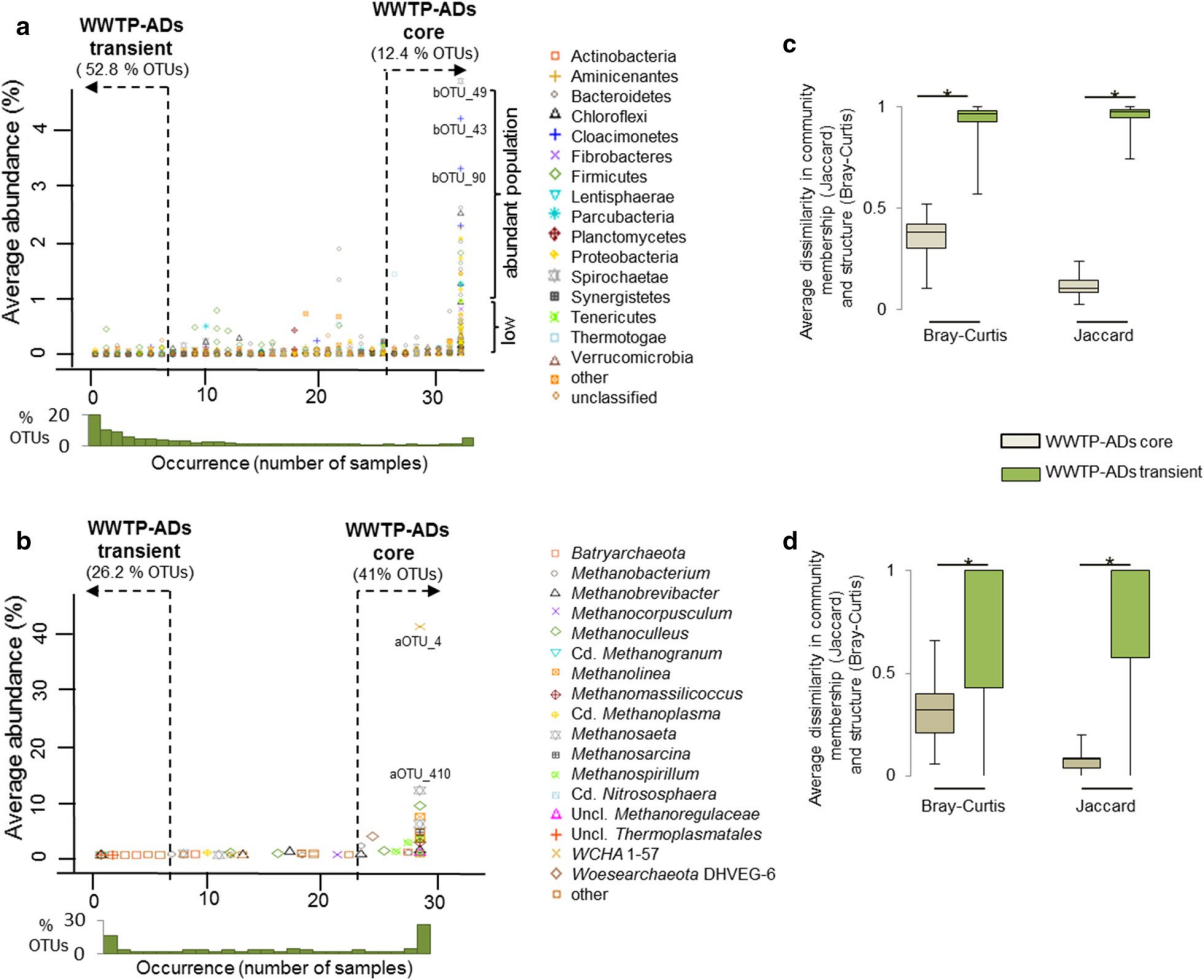
Average abundance versus occurrence plots (WWTP-ADs core and WWTP-ADs transient microbiomes) for **a** bacterial and **b** archaeal OTUs separately analysed from the WWTP-ADs. WWTP-ADs core OTUs were defined as appearing in over 80% of analysed WWTP sludge samples; WWTP-ADs transient OTUs were defined as being present in less than 20% of analysed WWTP samples. Density plots (green bars) displayed below the graphs **a** and **b** represent the percentage of OTUs occurring in a specified number of samples. Community structural stability (Bray–Curtis dissimilarity) and membership (Jaccard dissimilarity) for WWTP-ADs core and WWTP-ADs transient populations of OTUs for **c** bacteria and **d** archaea. Values range from 0 (identical) to 1 (100% different). The boxes represent the interquartile range and the error bars show the 95% confidence intervals. Differences between the core and transient communities for the calculated indices were significant (Wilcoxon signed rank test with continuity; *p* < 0.05)

Since the observed AD-core did not show any core characteristics for the studied WWTPs ADs, we evaluated these reactors separately. The resulting WWTP-ADs core _b_OTUs represented 12.4% of bacterial richness and accounted for 82.0% ± 6.2 of relative bacterial abundance (Fig. 5a). Their taxonomic composition was much different from the general AD-core and the majority of WWTP-ADs core _b_OTUs was assigned to *Proteobacteria* (103 _b_OTUs), *Bacteroidetes* (57 _b_OTUs), *Firmicutes* (36 _b_OTUs) and *Chloroflexi* (30 _b_OTUs). In terms of the relative abundance *Proteobacteria*, *Bacteroidetes* and *Cloacimonetes* were the dominant phyla. Out of the 342 WWTP-ADs core _b_OTUs, 24 represented candidate bacterial phyla and all together accounted for an average of 17.8% of the relative bacteria abundance in the studied WWTP ADs (Additional file 5: Table S5). There were only 27 _b_OTUs common to the general AD-core and WWTP-ADs core, mainly assigned to *Bacteroidetes*, *Firmicutes* and *Proteobacteria*; showing little species overlap between the two AD systems. Three most abundant (relative abundance ≥ 3%) WWTP-ADs core _b_OTUs were not AD-core, and were assigned to *Cloacimonetes* (_b_OTU-43 and _b_OTU-90) and one to *Spirochaetes* (_b_OTU-49). Around 89% of _b_OTUs (representing on average 1.44% ± 0.8 of the reads) were classified to WWTP-ADs transient _b_OTUs, and were mainly assigned to *Firmicutes* (328 OTUs), *Proteobacteria* (297 OTUs) and *Chloroflexi* (107 OTUs). Even though, according to [38] the major fraction of microbial populations found in WWTP-AD reactors is able to actively grow, we think that the occurrence of WWTP-ADs transient OTUs is rather related to the reactor feed residue and not to competitive metabolism. Interestingly, it has been shown for other WWTP-ADs that even some of the most abundant microbes (e.g. *Ca.* Microthrix) were related to influent streams as well [35]. In our study, three *Ca.* Microthrix _b_OTUs (_b_OTU-351, 882 and 1418) were assigned to the WWTP-ADs core; however, they represented the low abundant population of bacteria. *Ca. Microthrix* is a known aerobe and was shown to temporarily dominate aerobic tanks of the WWTPs in Luxembourg [47]. Regarding the relatively short sludge retention time and the fact that sewage-activated sludge is the only feeding substrate of the WWTP-AD reactors, it may happen that some of the WWTP-ADs core bacteria do not actively contribute to the AD process and they are present because they were fed into the reactor.

Archaeal core specific to WWTP-ADs was composed of 25 _a_OTUs (21 classified to *Euryarchaeota*, three to *Bathyarchaeota* and one to *Woesearchaeota*) and represented 97.9% ± 0.8 of the relative archaeal abundance per sample. Five WWTP-ADs core _a_OTUs were also designated AD-core OTUs, including _a_OTU-2 (*Methanoculleus* sp.), _a_OTU-1 (*Methanosarcina* sp.), _a_OTU-5 (*Methanomassiliicoccus* sp.), _a_OTU-48 (*Methanobacterium* sp.) and _a_OTU-3 (*Methanosaeta* sp.). Widespread in WWTP-ADs and the most abundant _a_OTU-4 (relative abundance in WWTP-ADs was 38.4% ± 10.5) assigned to an unclassified WSA2, was only present in half of the samples from agricultural and bio-waste treating AD units, and at much lower abundance (Fig. 2b). While there were no _a_OTUs representing the phylum *Bathyarchaeota* in the general AD-core, two were classified as WWTP-ADs core _a_OTUs (_a_OTU-6 and _a_OTU-15). Similar to the general AD-core, WWTP-ADs transient _a_OTUs were scarce in the reactors (0.1% ± 0.1 of the relative archaea abundance). Twenty _a_OTUs were neither core nor transient, and accounted for 2.1% ± 0.7 of the relative archaea abundance. They were mainly assigned to *Euryarchaeota*. Core archaeal population of WWTP ADs seemed more stable than the general AD-core (Fig. 5d). This might be related to the biogranules which form in WWTP systems, thus providing a protective microenvironment to the methanogens situated inside the granules; in comparison to the non-layered microbial distribution observed in non-WWTP AD systems [48].

### Abundance of candidate phyla in AD reactors

**Fig. 6:**
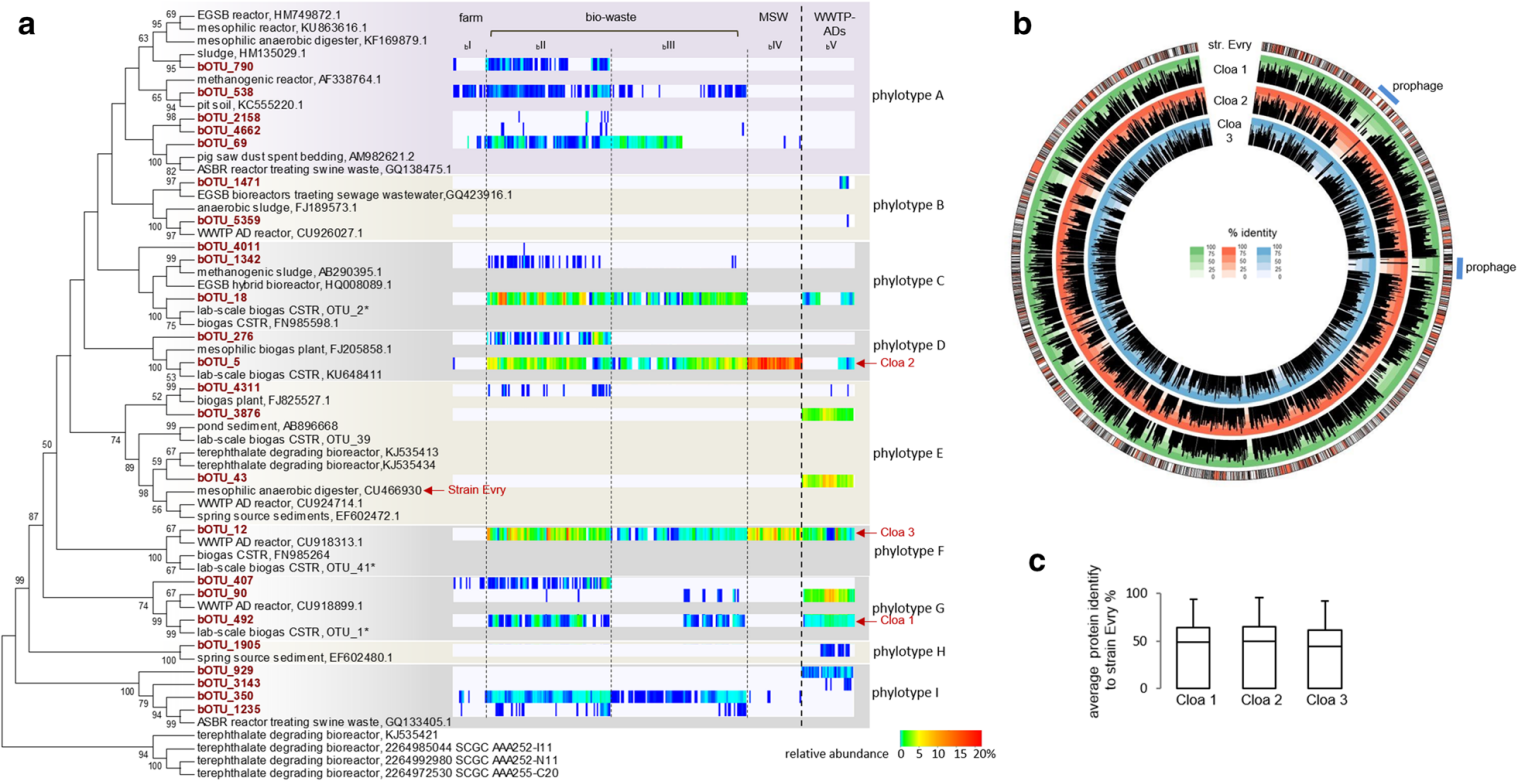
Neighbor-joining phylogenetic tree (**a**) based on partial 16S rRNA genes of *Cloacimonetes*_b_OTUs identified in this study and the comparison of the protein content for three newly reconstructed *Cloacimonetes* genomes (**b**, **c**). Bootstrap support values higher than 50% were displayed next to the nodes on the tree (**a**). Heat map representation of the relative _b_OTU abundance was calculated for each sample representing the time series of the year monitoring of the 20 AD reactors analysed in this study, and was organised by the cluster affiliation (separated by dashed vertical lines). Bold dashed vertical line separates the agricultural and bio-waste treating units from the WWTP-ADs. An asterix highlights the OTUs from the previous Goux et al. study [27]. Circular representation (**b**) of the *Candidatus* Cloacamonas acidaminovorans (strain Evry) protein content (circle one from the outside; hypothetical proteins are highlighted in red colour, block size corresponds to the protein size in aa), and its similarity to the respective homologous proteins in the metagenome-reconstructed genomes of three other *Cloacimonetes* species (Cloa 1, 2 and 3 corresponding, respectively, to the _b_OTUs-492, 5 and 12; three internal circles, respectively). Prophage regions identified in the genome of the strain Evry are highlighted with blue lines. Average protein similarity (for the homologous proteins) for Cloa 1, 2 and 3 to the *Candidatus* Cloacamonas acidaminovorans (strain Evry) proteins is shown on the graph **c**. The boxes represent the interquartile similarity range and the error bars show the 95% confidence intervals

In total, around 450 _b_OTUs were assigned to candidate bacterial phyla and their abundance differed significantly in the different AD systems. Their relative abundance was the lowest in farm reactors (on average 0.93% of bacterial community), followed by the bio-waste (7.56%) and WWTP-ADs (19.9%). Candidate phyla were the most abundant in a MSW-fed reactor (U-7), where they accounted for 21.15% of the whole bacterial community (mainly attributed to the dominance of *Cloacimonetes*_b_OTU-5). Five OTUs representing candidate phyla *Parcubacteria* (formerly known as OD1; 1.9% ± 0.8 average bacterial abundance) and two *Aminicenantes* (OP8; 2.5% ± 1.1 average bacterial abundance) formed part of the abundant WWTP-ADs core population of bacteria. Except for farm reactors, *Cloacimonetes* was dominant between the candidate phyla (from 60.1 to 89.8% of average abundance). *Cloacamonales* were the most abundant bacterial order in WWTP-ADs, but the dominant *Cloacimonetes* OTUs were different from those dominating in agricultural and bio-waste treating units (Fig. 6a). A neighbor-joining tree constructed using the 16S rRNA *Cloacimonetes* OTUs identified in this study and other phylogenetically similar sequences supported the existence of a high diversity within the *Cloacimonetes* phylum. Ten major phylotypes were distinguished, clearly separating _b_OTUs according to the anaerobic digestion system. Phylotype A grouped _b_OTUs that were dominating in agricultural and bio-waste treating units. Phylotypes B, E and H were mainly found in WWTP-ADs. *Cloacimonetes* phylotypes C, D, F, G and I were equally abundant in both anaerobic digestion systems. Such distribution of species could indicate the niche specialisation in the case of some *Cloacimonetes*, and it has not been discussed before in the literature.

*Cloacimonetes* were first discovered in a municipal WWTP in Evry, where they represented 85% of the generated clone library [49]. Preliminary analyses of their genomic content suggesting their involvement in cellulose degradation were further confirmed experimentally [50]. Moreover, reconstructed genomes of *Cloacimonetes* candidates contain most of the genes encoding enzymes involved in propionate oxidation via the methylmalonyl-CoA pathway, suggesting that they might be involved in a syntrophic oxidation of propionate [51]. Sequence-based homology search revealed that the partially reconstructed genomes corresponding to the *Claocimonetes*_b_OTU_5 (Cloa 1), _b_OTU-12 (Cloa 2) and _b_OTU-492 (Cloa 3) had 65.5% ± 3.9 (average protein identity below 50%) of their proteins similar to the *Candidatus* Cloacamonas acidaminovorans (strain Evry; Fig. 6b, c). This could indicate similar metabolisms. However, most (around 70%) of the non-homologous proteins (on average representing one-third of the protein pool) were assigned as hypothetical, thus functionally unknown. The putative prophages were also missing. Due to the lack of isolates, it is, therefore, impossible based on a simple genome comparison, to discuss the differences between the metabolic capabilities of the proposed *Cloacimonetes* phylotypes. However, according to our previous bio-augmentation success with *Cloacimonetes*-enriched sludge [52], we think *Cloacimonetes* might be one of the keystone species that according to the concept of the centres of concentrated activity [42] might take a central role in the anaerobic digestion process.

Candidate WSA2 archaeon dominated the WWTP ADs representing 38.4% ± 10.3 of archaeal population in these reactors. Recent phylogenetic analyses indicate that this group is different from other *Euryarchaeota*, and reconstructed genome suggests WSA2 to be the first methanogen restricted to methanogenesis through methylated thiol reduction [53]. Using acetate, malonate and especially propionate as carbon source, it could be regarded as a beneficial microbe to the stability of the AD process (i.e. increased structural stability of WWTP-core archaea towards general AD-core might be related to the presence of these archaea), as propionate tends to accumulate under sub-optimal conditions in AD reactors [27]. Moreover, using the methylated thiol reduction, WSA2 may potentially compete with sulfate reducers thus controlling the amount of the produced H_2_S in the reactor.

### Core microbiomes in a global context

Taking into consideration the different assay designs (e.g. different primer pair used, handling of sampled material, thresholds used during the bioinformatics analyses, etc.) undertaken in different studies it is difficult to directly compare the results from the literature and verify the universality of the proposed core community concept in different digestion systems worldwide. Even if 16S rRNA do not reassemble well in metagenomes, as preliminary analysis, we blasted the 16S rRNA sequences generated in this study over the 8000 assembled genomes recently reconstructed from 1500 public metagenomes [54]. As a result, 123 OTUs matched with at least 97% identity to 103 unique genomes (Additional file 8: Table S7). Most of them corresponded to the transient OTUs, and only eight genomes represented AD-core OTUs and 31 WWTP-ADs core bacteria. Therefore, further sequencing efforts and cultivation studies are necessary, especially with regards to the microbes present in agricultural and bio-waste treating AD reactors (largely underrepresented in public databases) to better uncover the real metabolic capabilities of the AD-core microbes.

Still, some of the dominant AD-core bacteria described here (e.g. _b_OTU-9 and _b_OTU-4) were previously discovered in other ADs, including thermophilic anaerobic digesters in Japan [55] or CSTR bioreactors in Germany [56], suggesting their worldwide distribution. In the case of the agricultural biogas plants in Germany, more than 75% of reactors utilise maize as a major feeding substrate [57]. Therefore, we could expect the bacterial cores in these reactors to be less diverse than our AD-core, as they have specialised in processing mainly this explicit biomass. In Luxembourg and Belgium, most of the reactors receive a very complex and highly diversified along the year feed (e.g. MSW), therefore, we believe that the diverse microbial communities that have established might be considered more representative of the different anaerobic digestion systems worldwide.

## Conclusions

Globally, the growing appreciation of understanding microbial ecology to improve the stability and the efficiency of full-scale bioenergy production systems encourages researchers to study the AD microbial communities in a profound manner. In this study, by monitoring 20 AD reactors over a period of 1 year, we demonstrated that the diversity of microbial communities was high and varied significantly between differentially operated AD reactors, mainly due to the presence of highly diverse and dynamic transient _b_OTUs. However, using the core community concept, we have shown that the ubiquitously distributed OTUs (roughly 2.5 and 12.4% of bacterial and archaeal OTUs, respectively), accounted for over 70% of the community abundance in agricultural and bio-waste treating units. Regardless the reactor operational conditions and feedstock composition (i.e. agricultural waste, manure, MSW, etc.), core communities were very stable over the year in terms of community membership and species dynamics. Proper installation design (e.g. U-7) has proved that even the microbial communities fed with highly diversified MSW might be very stable, opening the doors for an increased MSW valorisation in AD reactors. WWTP-ADs were shown to operate with distinct microbiomes where 12.4% of bacterial and 41% of archaeal OTUs were classified as a WWTP-ADs core. Based on our results, we hypothesize that the core microbial community of an AD process might have a more reduced complexity in terms of species richness than previously expected. Indeed, according to the “insurance hypothesis” [31], the greater the diversity of the species responses, the lower the species richness at which the ecosystem becomes redundant. In this sense, future research should prioritise the characterisation of the largely unknown and often representing candidate phyla AD-core and WWTP-ADs core microbes to unravel the complexity of their metabolic pathways and their real potential in a methanogenic reactor [58]. Characterisation of these putative key process microbes and a better comprehension of the interactions between them will help to establish the relation between species diversity (composition) and community functionality and dynamics. This would allow us to understand whether highly diverse communities with the portfolio of serial pathways (continuous competition between highly diverse species can; however, destabilise the community structure) or less diverse microbiomes with complementarity of functions (lower species competition should assure better structural stability of the ecosystem) are better adapted to a high and stable industrial biogas production.

## Methods

### AD samples

AD reactors located at the wastewater treatment plants correspond to Petange (P), Schifflange (S) and Bettembourg (BT) units. U-7 is a MSW-valorising plant located in Mondercange. The remaining studied reactors are private installations. After thorough reactor mixing and careful purging of the sampling port, a representative sludge volume of 2 L was subsampled from each reactor and aliquots were frozen on site in liquid nitrogen. Back to the laboratory, frozen aliquots were stored at − 80 °C prior to the analysis. TS (24 h at 105 °C) and VS (6 h at 550 °C) were determined in the remaining sludge samples according to the 4630 VDI norm [59]. The pH was measured with a pH 196 Microprocessor pH meter connected to a SenTix^®^ 21 pH electrode (WTW, Weilheim, Germany). TIC and NH_4_–N were measured in conformity with the manufacturer’s protocol, using the BiogasPro system (RIMU, Königsbrunn, Germany). The concentration of VFAs was measured following an ether extraction and using a gas chromatography (Agilent technologies, Santa Clara, USA) equipped with a Varian CP-FFAP column and a flame ionization detector (FID). The migration was done with helium (He) as a carrier gas. The total VFAs concentrations (mg kg^−1^) were expressed as the sum of the individual VFA concentrations measured for acetate, propionate, isobutyrate, butyrate, isovalerate, valerate and caproate. Genomic DNA was extracted from the sludge quantity of 200–500 mg, with the PowerSoil DNA Isolation kit (MoBio) according to the manufacturer’s protocol.

### Selection of amplification primers and sequencing assay design

Based on the in silico results [60], we pre-selected two primer pairs to separately target bacterial and archaeal 16S rRNA gene (Table 2). The Illumina platform-compatible dual-index paired-end approach was designed analogously to the approaches designed elsewhere ([61], Illumina 16S Metagenomic Sequencing Library Preparation Note Part # 15044223 Rev.A), with additional modifications. Each forward and reverse primer consisted of an Illumina-compatible forward/reverse primer overhang attached to the 5′ end. Additionally, a heterogeneity spacer of four degenerate nucleotides (Ns) was added to the forward primer, between the primer overhang and the locus-specific sequence. The Illumina barcodes and sequencing adapters were added during the subsequent cycle-limited amplification step using Nextera XT Index kit (Illumina).

**Table 2:** Primers pairs used in this study to specifically amplify 16S rRNA genes from bacteria and archaea

Domain	Primer name	Sequence 5′→3′	Variable region	Average amplicon size (bp)	In silico specificity^a^	Ref.
Bacteria	S-D-Bact-0909-a-S-18	ACTCAAAKGAATWGACGG	V6–V8	484	0–78.8% 1–89.6%	[60]
	S-*-Univ-1392-a-A-15	ACGGGCGGTGTGTRC				
Archaea	S-D-Arch-0519-a-S-15	CAGCMGCCGCGGTAA	V4–V6	526	0–79.3% 1–93.7%	[60]
	S-D-Arch-1041-a-A-18	GGCCATGCACCWCCTCTC				

^a^The in silico specificity against the rRNA gene sequences deposited in SILVA database v132; the first number refers to the number of mismatches, while the percentage refers to the proportion of taxa that was targeted specifically

### Preparation of small rRNA amplicon libraries and sequencing on Illumina platform

The small rRNA amplicons were generated using the Q5 Hot Start High-Fidelity DNA Polymerase (New England Biolabs Inc.) in triplicate 25-µL reactions, using 1 ng of template DNA, 0.4 µM of each primer and BSA (Sigma) at the final concentration of 1 mg/mL. The reaction mixtures were subjected to an initial denaturation at 98 °C for 30 s, followed by 22–26 cycles at 98 °C for 10 s, annealing for 30 s and elongation at 72 °C for 30 s, and a final elongation at 72 °C for 2 min (Additional file 3: Table S2). Following the amplification, the triplicate amplifications were visualized on 3% agarose gels, pooled together and purified with the AMpure magnetic beads (Agencourt) and quantified with the Qubit dsDNA HS assay kit (Life Technologies). The concentration of the amplicons was adjusted to 1 ng/µL and 1 µL of each library was used as a template in a second PCR reaction where the Nextera XT barcodes and the Illumina adapters necessary for hybridization to the flow cell were added. PCR amplification was performed with the same enzyme and cycling conditions were as described above, with the total number of cycles reduced to eight and an annealing temperature of 55 °C. The resulting amplicons were purified with the AMpure magnetic beads (Agencourt), quantified and pooled in equimolar concentrations (between 96 and 384 samples were mixed and sequenced in a single sequencing run). The detailed protocols are provided as supporting information (Additional file 3). The final concentration of the library pool was determined with a KAPA SYBR FAST Universal qPCR Kit (Kapa Biosystems), according to the manufacturer’s instructions. Libraries were mixed with Illumina-generated PhiX control library, denatured with fresh NaOH, diluted to a final concentration of 8 pM, and sequenced with the MiSeq Reagent Kit V3-600 cycles (LIST, Luxembourg), using the sequencing primers for reads 1–4 provided with the kit.

### Sequencing data analysis and statistics

The obtained small rRNA amplicon sequence reads were de-multiplexed, quality trimmed and OTUs were constructed with UPARSE version v7.0.1090 [62], and taxonomically annotated using the non-redundant SILVA SSU ribosomal database v.128 [63]. Final nucleotide sequences (OTUs) were deposited in the GenBank database under the accession numbers KU648407–KU659020.

All diversity analyses were performed on per sample subsampled shared files (normalize.shared command) using mothur v.1.34.4 or later [64]. Community richness and diversity were calculated using sobs and invsimpson calculators, respectively. Community evenness was measured via the invsimpsoneven coefficient on a normalised scale from 0 (uneven; one or several dominant OTUs and many singlets) to 1 (perfectly even). The stability of community structure and membership was calculated using the Bray–Curtis dissimilarity and Jaccard indices. Average species abundance was calculated excluding the samples where OTU was not represented by at least one read. Statistical significance of data was calculated using either Wilcoxon signed rank test or Kruskal–Wallis test, or ANOSIM, and the differences were considered statistically significant at *p* value ≤ 0.05. NMDS analyses and heat maps were done using R version 3.4.0 [65]. Partial 16S rRNA gene sequences for the *Cloacimonetes*_b_OTUs and the close relatives were aligned using MAFFT algorithm [66] and the neighbor-joining phylogenetic tree was constructed with MEGA6 [67].

The CCA analyses were performed with the CANOCO software version 4.5 [68] and the significance test was done using Monte Carlo permutation (500 times). Spearman correlations were calculated using R version 3.4.0 [65], and the calculated correlations were considered statistically significant at *p* value ≤ 0.05. Three metagenome reconstructed genomes of novel *Cloacimonetes* (Cloa 1, 2 and 3) were taken from Broeksema et al. [28] and correspond to bins 1, 31 and 13, respectively, in that study. The similarity of their protein content towards the sequenced genome of the *Candidatus Cloacamonas acidaminovorans* [24] was visualized with Circos [69].

## Additional files

**Additional file 1: Figure S1.** Characterisation of the studied AD reactors. Typical design of (A) PFR-type (U-7) and (B) CSTR-type reactors (all other units) of the studied biogas plant installations.

**Additional file 2: Table S1.** Characterisation of the studied AD reactors. Detailed (per sample) characterisation of the studied AD reactors.

**Additional file 3: Figure S2.** The 16S rRNA gene amplicon assay design. Design of amplification primers used in this study; first- and second-level barcoding strategy, and **Table S2**. The 16S rRNA gene amplification conditions (1st PCR).

**Additional file 4: Table S3.** The 16S rRNA gene amplicon assay validation. Letter codes for the additional libraries prepared for six samples S1–S6 used to validate the second-level barcoding strategy; **Table S4**. Different preparations of libraries for six samples S1–S6 used to validate the second-level barcoding strategy; **Figure S3**. NMDS of pairwise Bray–Curtis distance comparisons calculated for separate and mix amplicon library preparations (intra-DNA extraction comparison) and for the two DNA extractions tested (inter-DNA extraction comparison) for six selected samples (S1–S6); **Figure S4**. Taxonomical distribution of sequencing reads for separate and mix amplicon library preparations for the two different DNA extractions (Ext1 and Ext2) tested for six selected samples (S1–S6); **Figure S5**. Median richness (sobs index) and diversity (invsimpson index) metrics calculated for separate and mix amplicon library preparations and for the two different DNA extractions (Ext1 and Ext2) tested for six selected samples (S1–S6).

**Additional file 5: Table S5.** The 16S rRNA gene amplicon sequencing results of 1-year monthly time series of 20 AD reactors located in Luxembourg and Belgium. Relative abundance (%, normalised number of reads) and taxonomic affiliation of the 16S rRNA amplicon sequencing OTUs for bacteria and archaea.

**Additional file 6: Figure S6.** The 16S rRNA gene amplicon sequencing results of 1-year monthly time series of 20 AD reactors located in Luxembourg and Belgium. Rarefaction curves based on the calculated species richness (Sobs) for bacteria (A) and archaea (B). Rank abundance curve of bacterial OTUs (C); **Figure S7.** Median bacterial richness (A, Sobs), diversity (B, invsimpson) and evenness (C, invsimpsoneven) per reactor during one-year monitoring survey; **Figure S8.** Cluster-specific average Bray–Curtis dissimilarity in community structures and Jaccard (Jclass) dissimilarity in community membership for bacteria (A, B) and archaea (C, D) for each of the community clusters; **Figure S9**. Median archaeal richness (A, Sobs), diversity (B, invsimpson) and evenness (C, invsimpsoneven) per reactor during 1-year monitoring survey; **Figure S10**. Calculated average Bray–Curtis dissimilarity in “AD-core” and “AD-transient” community structures and Jaccard dissimilarity in “AD-core” community and “AD-transient” membership for bacteria (A, B) and archaea (C, D) for each of the studied reactors.

**Additional file 7: Table S6.** The 16S rRNA gene amplicon sequencing results of 1-year monthly time series of 20 AD reactors located in Luxembourg and Belgium. Spearman correlation matrix between operational data and dominant microbial groups/ OTUs.

**Additional file 8: Table S7.** The 16S rRNA gene amplicon sequencing results of 1-year monthly time series of 20 AD reactors located in Luxembourg and Belgium. Blast search results for _b_OTUs analysed in this study against the 8000 metagenome-reconstructed genomes from the study by Parks et al. [54].
