# Supplementary material for: A year of monitoring 20 mesophilic full-scale bioreactors reveals the existence of stable but different core microbiomes in bio-waste and wastewater anaerobic digestion systems
Source: Biotechnol Biofuels. 2018 Jul 19;11:196. doi: 10.1186/s13068-018-1195-8 (PMC6052691; doi:10.1186/s13068-018-1195-8)
Supplement: Supplementary file 1 — Additional file 1: Figure S1. Characterisation of the studied AD reactors. Typical design of (A) PFR-type (U-7) and (B) CSTR-type reactors (all other units) of the studied biogas plant installations. [file 13068_2018_1195_MOESM1_ESM.doc]

**Additional file 1: Characterisation of the studied AD reactors**


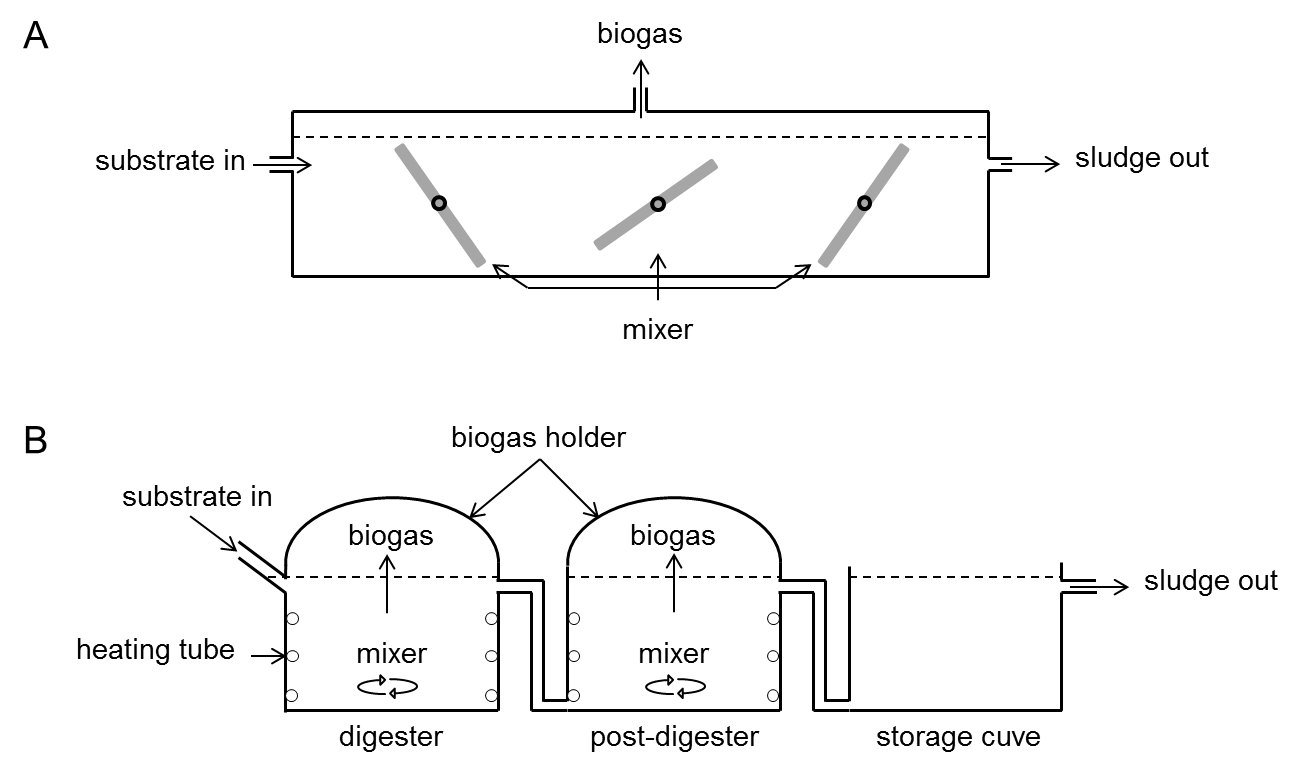


**Figure** **S1**

Typical design of (A) PFR-type (U-7) and (B) CSTR-type reactors (all other units) of the studied biogas plant installations.
