# Supplementary material for: A year of monitoring 20 mesophilic full-scale bioreactors reveals the existence of stable but different core microbiomes in bio-waste and wastewater anaerobic digestion systems
Source: Biotechnol Biofuels. 2018 Jul 19;11:196. doi: 10.1186/s13068-018-1195-8 (PMC6052691; doi:10.1186/s13068-018-1195-8)
Supplement: Supplementary file 3 — Additional file 3: Figure S2. The 16S rRNA gene amplicon assay design. Design of amplification primers used in this study; first- and second-level barcoding strategy, and Table S2. The 16S rRNA gene amplification conditions (1st PCR). [file 13068_2018_1195_MOESM3_ESM.doc]

**Additional file 3: The 16S rRNA gene amplicon assay design**

Different universal primer pairs targeting bacteria and archaea were initially tested, however each time there was a strong bias towards bacteria, largely underestimating the diversity of archaea (data not shown). Therefore specific primer pairs were used to separately amplify bacterial and archaeal 16S rRNA during the 1^st^ PCR round (Table 2). The in silico specificity of the selected primer pairs was verified using the SILVA SSU ribosomal database v.128. With no mismatch the bacterial primer pair was targeting respectively 78.8 % of all known bacterial and 0 % of the archaeal 16S rRNA gene sequences, thus showing to be bacteria-specific. The archaeal primer pair was targeting respectively 79.3 % of all known archaeal and 0 % of the bacterial 16S rRNA gene sequences, thus showing to be archaea-specific. Selected primer pairs were also tested using two different mock communities (a mixture of gDNA extracted from species with sequenced genomes and representing different microbial phyla commonly found in AD reactors), and showed to accurately amplify target sequences (data not shown; can be shared upon request). Additionally, domain-specificity of the selected primer pairs was confirmed at the level of the sequencing results, where less than 1 % of reads were domain-unspecific. Each forward and reverse primer, in addition to the locus-specific sequence, contained in its 5´ end specific Illumina-compatible primer overhangs to which Illumina barcodes were attached during the second PCR (Figure S2). Each forward primer contained a heterogeneity spacer of four degenerate nucleotides (N) in order to increase the nucleotide diversity. At the time the study was conducted, only 96 different barcode combinations were available from the supplier (currently 384). Therefore to maximize the number of samples sequenced on the same flow cell (without the need to design additional custom primers), a second-level barcoding was evaluated (2^nd^ level barcoding; Figure S2). Moreover, to maximize the effective length of the MiSeq Reagent V3-600, gene products of a size range between 484 and 526 bp were targeted. Following the 1^st^ PCR, bacterial and archaeal amplicons of the same sample were pooled together and labelled with the same Illumina barcode during the 2^nd^ PCR. Following the sequencing run, different samples were identified based on the attached Illumina dual barcodes (1^st^ level barcoding), while to separate bacterial and archaeal reads we used the sequences of the respective locus-specific primers as barcodes (2^nd^ level barcoding; Figure S2). Maximizing the number of samples in a sequencing run and minimizing the amount of added control genomic DNA from the phage PhiX to an average of 4.2 % ± 3.1, allowed reducing the cost. Furthermore, combining different types of amplicons in a single run allowed increasing the heterogeneity of samples in a pool, what resulted in improved sequencing quality. Furthermore, to minimize the PCR biases we incorporated many of the known wet bench practices, including: (1) the total number of PCR cycles reduced to the minimum (Table S2), (2) the use of a proofreading polymerase and (3) pooling triplicate PCR reactions together prior sequencing.

**
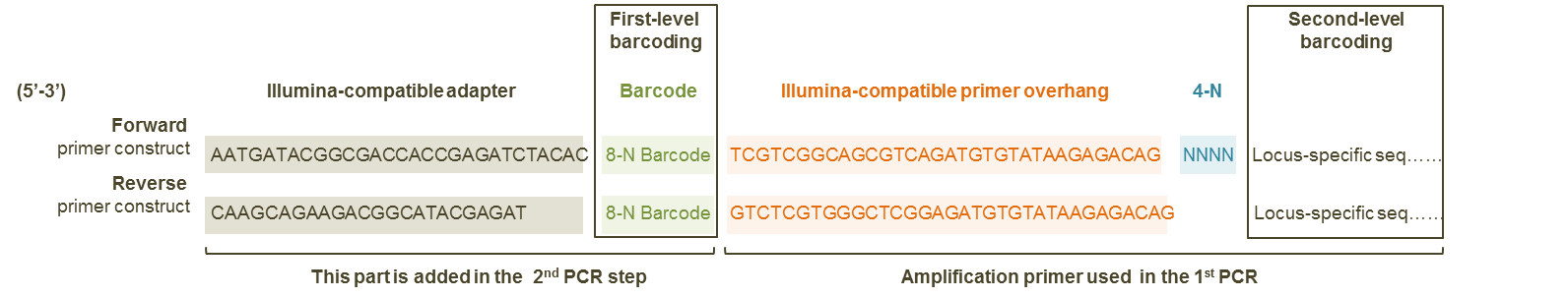
**

**Figure S2**

Design of amplification primers used in this study; first- and second-level barcoding strategy.

**Table S2**

The 16S rRNA gene amplification conditions (1^st^ PCR).

| Domain | Initial denaturation | Denaturation | Annealing | Elongation | Number of cycles | Final elongation |
| --- | --- | --- | --- | --- | --- | --- |
| Bacteria | 98 °C  30 s | 98 °C  10 s | 58 °C 30 s | 72 °C  30 s | 22 | 72 °C  2 min. |
| Archaea |  |  | 65 °C 30 s |  | 26 |  |
