# Supplementary material for: A year of monitoring 20 mesophilic full-scale bioreactors reveals the existence of stable but different core microbiomes in bio-waste and wastewater anaerobic digestion systems
Source: Biotechnol Biofuels. 2018 Jul 19;11:196. doi: 10.1186/s13068-018-1195-8 (PMC6052691; doi:10.1186/s13068-018-1195-8)
Supplement: Supplementary file 4 — Additional file 4: Table S3. The 16S rRNA gene amplicon assay validation. Letter codes for the additional libraries prepared for six samples S1–S6 used to validate the second-level barcoding strategy; Table S4. Different preparations of libraries for six samples S1–S6 used to validate the second-level barcoding strategy; Figure S3. NMDS of pairwise Bray–Curtis distance comparisons calculated for separate and mix amplicon library preparations (intra-DNA extraction comparison) and for the two DNA extractions tested (inter-DNA extraction comparison) for six selected samples (S1–S6); Figure S4. Taxonomical distribution of sequencing reads for separate and mix amplicon library preparations for the two different DNA extractions (Ext1 and Ext2) tested for six selected samples (S1–S6); Figure S5. Median richness (sobs index) and diversity (invsimpson index) metrics calculated for separate and mix amplicon library preparations and for the two different DNA extractions (Ext1 and Ext2) tested for six selected samples (S1–S6). [file 13068_2018_1195_MOESM4_ESM.doc]

**Additional file 4: The 16S rRNA gene amplicon assay validation**

To validate the 2^nd^-level barcoding strategy and to evaluate the influence of the 2^nd^ PCR and the sequencing itself on the quality and the read distribution into bacterial and archaeal reads, when sequenced under the same barcode, we prepared additional libraries (Tables S3 and S4; Figures S3 – S5) for six samples (S1 – S6) representing different reactor feeding regimes: (1) wastewater AD digesters (S1 and S2), (2) on-farm reactors fed mainly with agricultural residues (S3 and S4), (3) municipal and green-waste (mostly garden and park residues) treating reactors (S5), and (4) reactors treating a mixture of agricultural residues (mainly manure) and bio-waste (S6). Two different DNA extractions (Ext1 and Ext2) were used to prepare separate libraries for each primer pair as well as to prepare mixed PCR amplicon libraries that were sequenced in duplicate or triplicate (technical replicates gave not significantly different results). Then, 16S rRNA amplicons for bacteria and archaea for the same sample and the same extraction were either sequenced under the same barcode or under different barcodes. Based on the calculated Bray-Curtis dissimilarity indices (Figure S3), there was no significant difference in the structure of bacterial and archaeal communities between the different libraries (libraries prepared separately for the different primers and sequenced under different barcodes and libraries for the two amplicon types mixed before the 2^nd^ PCR and sequenced under the same barcode) for the same sample and a single DNA extraction. This indicated that neither the second barcoding strategy nor the use of the different barcodes influenced negatively the sequencing results. However, the differences between the replicate libraries prepared for the two separate DNA extractions were statistically significantly different for several of the tested samples (Figure S3). At the same time, the pairwise comparison of the taxonomic distribution of reads for the different conditions tested and for the two DNA extractions for S1-S6, showed very similar trends (Figure S4). The calculated diversity and richness indices were also not significantly different for bacteria and archaea and for most of the samples, as further observed by small box plots and short whiskers (Figure S5).

**Table S3**

Letter codes for the additional libraries prepared for six samples S1 – S6 used to validate the second-level barcoding strategy

| Sample No | DNA extraction | Second-level barcoding | Targeted domain | Technical replicate |
| --- | --- | --- | --- | --- |
| **S1** to **S6** | **(1)** – 1st DNA extraction  **(2)** – 2nd DNA extraction | **mix** | **B** - Bacteria  **A** - Archaea | **1** to **3** |

Examples: S1(1)mix1 – sample1, first DNA extraction, mixed amplicons for bacteria and archaea, technical replicate 1; S2(2)B1 – sample 2, second DNA extraction, amplicons for bacteria, technical replicate 1.

**Table S4**

Different preparations of libraries for six samples S1 to S6 used to validate the second-level barcoding strategy. Letter code is as shown in Table S3.

| **Sample** |  | **S1** | **S2** | **S3** | **S4** | **S5** | **S6** |
| --- | --- | --- | --- | --- | --- | --- | --- |
| **Reactor feeding regime*** |  | 1 | 1 | 2 | 2 | 3 | 4 |
| **DNA extraction_1** | | | | | | | |
|  |  | S1(1)A | S2(1)A1 to 3 | S3(1)A | S4(1)A | S5(1)A | S6(1)A1 to 3 |
|  |  | S1(1)B | S2(1)B1 to 3 | S3(1)B | S4(1)B | S5(1)B | S6(1)B1 to 3 |
|  |  | S1(1)mix1 | S2(1)mix1 | S3(1)mix1 | S4(1)mix1 | S5(1)mix1 | S6(1)mix1 |
|  |  | S1(1)mix2 | S2(1)mix2 | S3(1)mix2 | S4(1)mix2 | S5(1)mix2 | S6(1)mix2 |
|  |  | S1(1)mix3 | S2(1)mix3 | S3(1)mix3 | S4(1)mix3 | S5(1)mix3 | S6(1)mix3 |
| **DNA extraction_2** | | | | | | | |
|  |  | S1(2)A | S2(2)A1 to 3 | S3(2)A | S4(2)A | S5(2)A | S6(2)A1 to 3 |
|  |  | S1(2)B | S2(2)B1 to 3 | S3(2)B | S4(2)B | S5(2)B | S6(2)B1 to 3 |
|  |  | S1(2)mix1 | S2(2)mix1 | S3(2)mix1 | S4(2)mix1 | S5(2)mix1 | S6(2)mix1 |
|  |  | S1(2)mix2 | S2(2)mix2 | S3(2)mix2 | S4(2)mix2 | S5(2)mix2 | S6(2)mix2 |
|  |  | S1(2)mix3 | S2(2)mix3 | S3(2)mix3 | S4(2)mix3 | S5(2)mix3 | S6(2)mix3 |

*(1) wastewater AD digesters; (2) on-farm reactors fed mainly with agricultural residues; (3) municipal and green-waste (mostly garden and park residues) treating reactors; and (4) reactors treating a mixture of agricultural residues (mainly manure) and bio-waste.

**
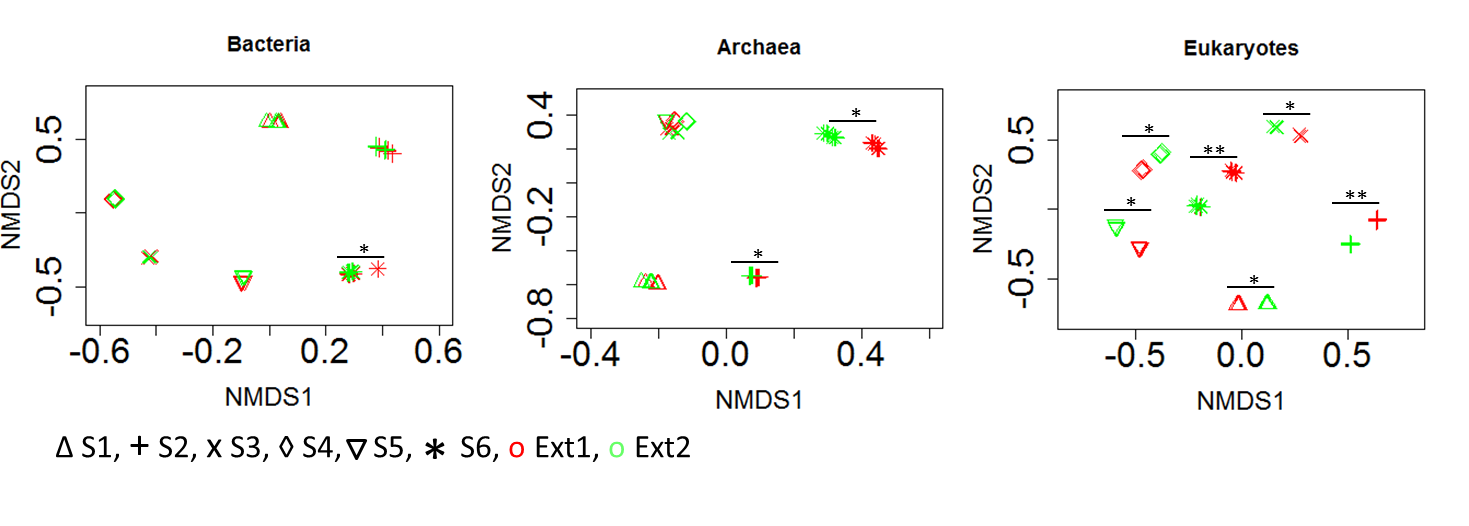
**

**Figure S3**

NMDS of pairwise Bray-Curtis distance comparisons calculated for separate and mix amplicon library preparations (intra-DNA extraction comparison) and for the two DNA extractions tested (inter-DNA extraction comparison) for six selected samples (S1-S6). Results are given for the bacteria-specific primer pair (stress 0.17, R^2^ 0.88) and archaea-specific primer pair targeting 16S rRNA gene (stress 0.05, R^2^ 0.99). Inside Ext1 and Ext2 (separate DNA extractions) different amplicon library preparations (replicates of separate and mix amplicon library preparations sequenced under different barcodes) were not significantly different for the two primer pairs tested (ANOSIM p > 0.05 and not indicated on the graph). Inter-DNA extraction results for the different amplicon library preparations and for the two different primer pairs tested were considered different for ANOSIM p ≤ 0.05 (*), and are indicated on the relative graphs.

**
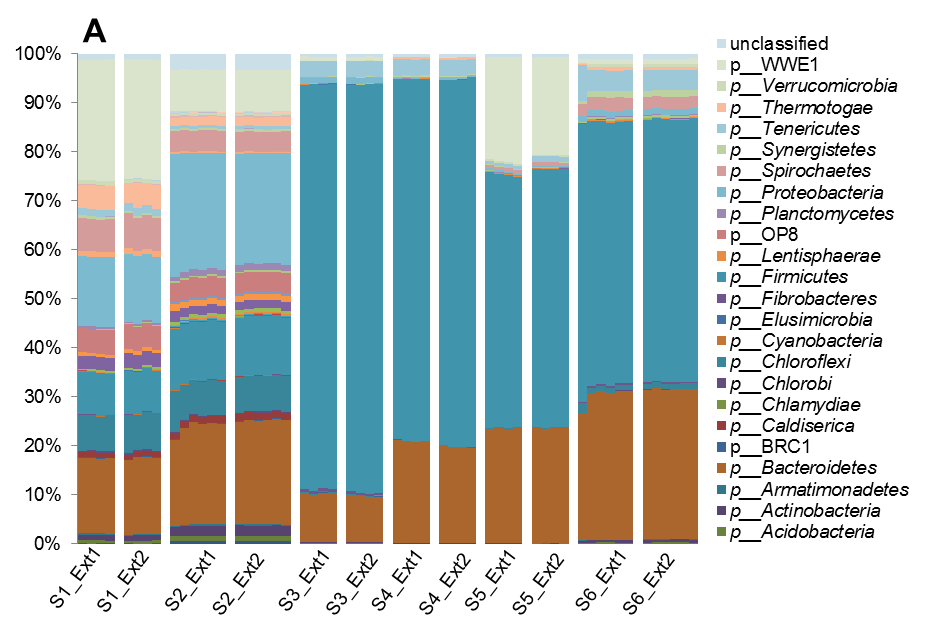
**

**
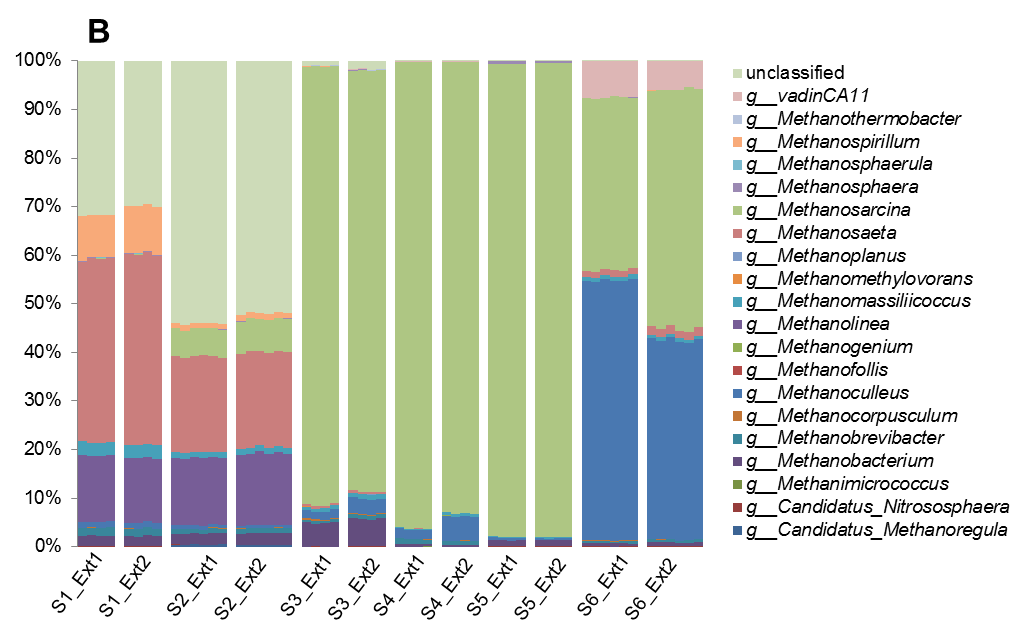
**

**Figure** **S4**

Taxonomical distribution of sequencing reads for separate and mix amplicon library preparations for the two different DNA extractions (Ext1 and Ext2) tested for six selected samples (S1-S6) using (A) bacteria-specific 16S rRNA gene primer pair (displayed at the phylum level for the major phyla); (B) archaea-specific 16S rRNA gene primer pair (displayed at the genus level). For sample name code, please see Table S4.


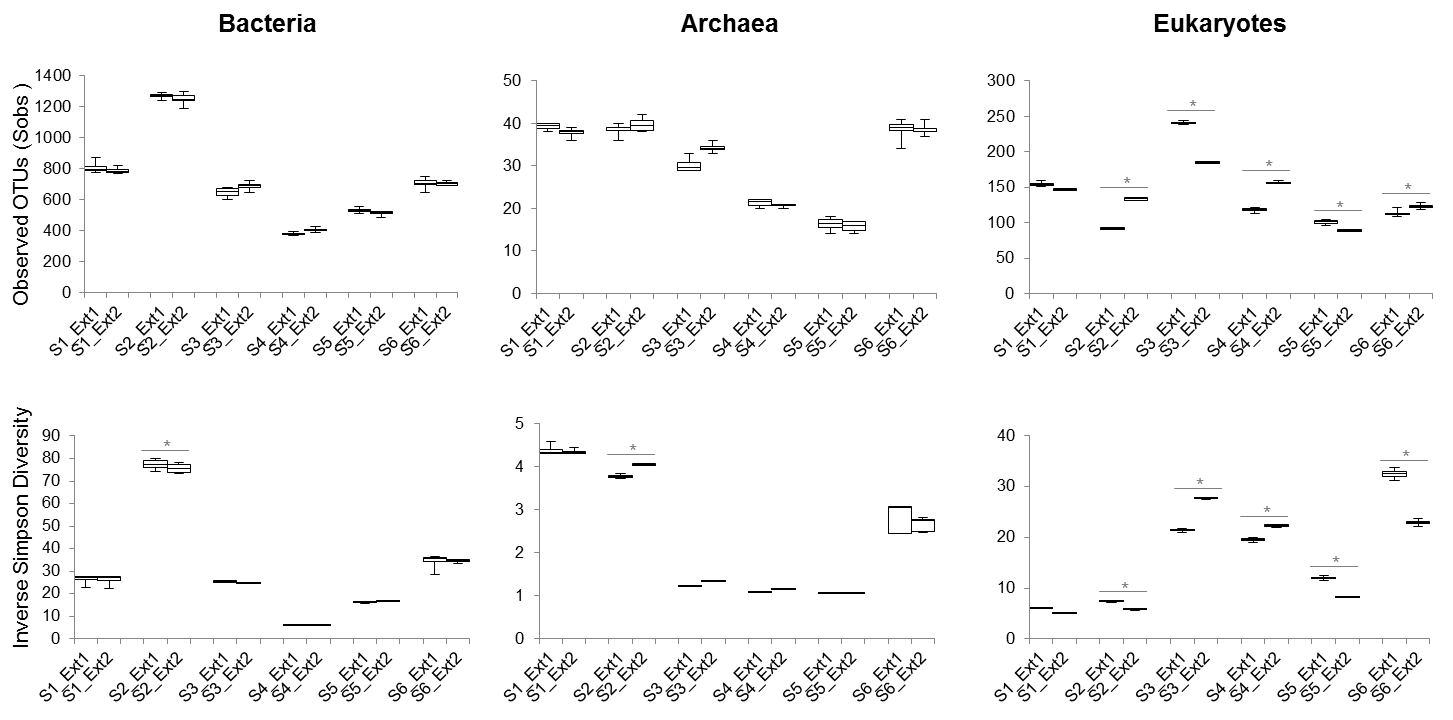


**Figure S5**

Median richness (sobs index) and diversity (invsimpson index) metrics calculated for separate and mix amplicon library preparations (Table S4) and for the two different DNA extractions (Ext1 and Ext2) tested for six selected samples (S1-S6). Statistical analyses were performed using Wilcoxon signed-rank test; differences were considered significant for p ≤ 0.05 (indicated with an asterisk).
