# Supplementary material for: A year of monitoring 20 mesophilic full-scale bioreactors reveals the existence of stable but different core microbiomes in bio-waste and wastewater anaerobic digestion systems
Source: Biotechnol Biofuels. 2018 Jul 19;11:196. doi: 10.1186/s13068-018-1195-8 (PMC6052691; doi:10.1186/s13068-018-1195-8)
Supplement: Supplementary file 6 — Additional file 6: Figure S6. The 16S rRNA gene amplicon sequencing results of 1-year monthly time series of 20 AD reactors located in Luxembourg and Belgium. Rarefaction curves based on the calculated species richness (Sobs) for bacteria (A) and archaea (B). Rank abundance curve of bacterial OTUs (C); Figure S7. Median bacterial richness (A, Sobs), diversity (B, invsimpson) and evenness (C, invsimpsoneven) per reactor during one-year monitoring survey; Figure S8. Cluster-specific average Bray–Curtis dissimilarity in community structures and Jaccard (Jclass) dissimilarity in community membership for bacteria (A, B) and archaea (C, D) for each of the community clusters; Figure S9. Median archaeal richness (A, Sobs), diversity (B, invsimpson) and evenness (C, invsimpsoneven) per reactor during 1-year monitoring survey; Figure S10. Calculated average Bray–Curtis dissimilarity in “AD-core” and “AD-transient” community structures and Jaccard dissimilarity in “AD-core” community and “AD-transient” membership for bacteria (A, B) and archaea (C, D) for each of the studied reactors. [file 13068_2018_1195_MOESM6_ESM.doc]

**Additional file 6: The 16S rRNA gene amplicon sequencing results of one-year monthly time series monitoring of 20 AD reactors located in Luxembourg and Belgium**

**
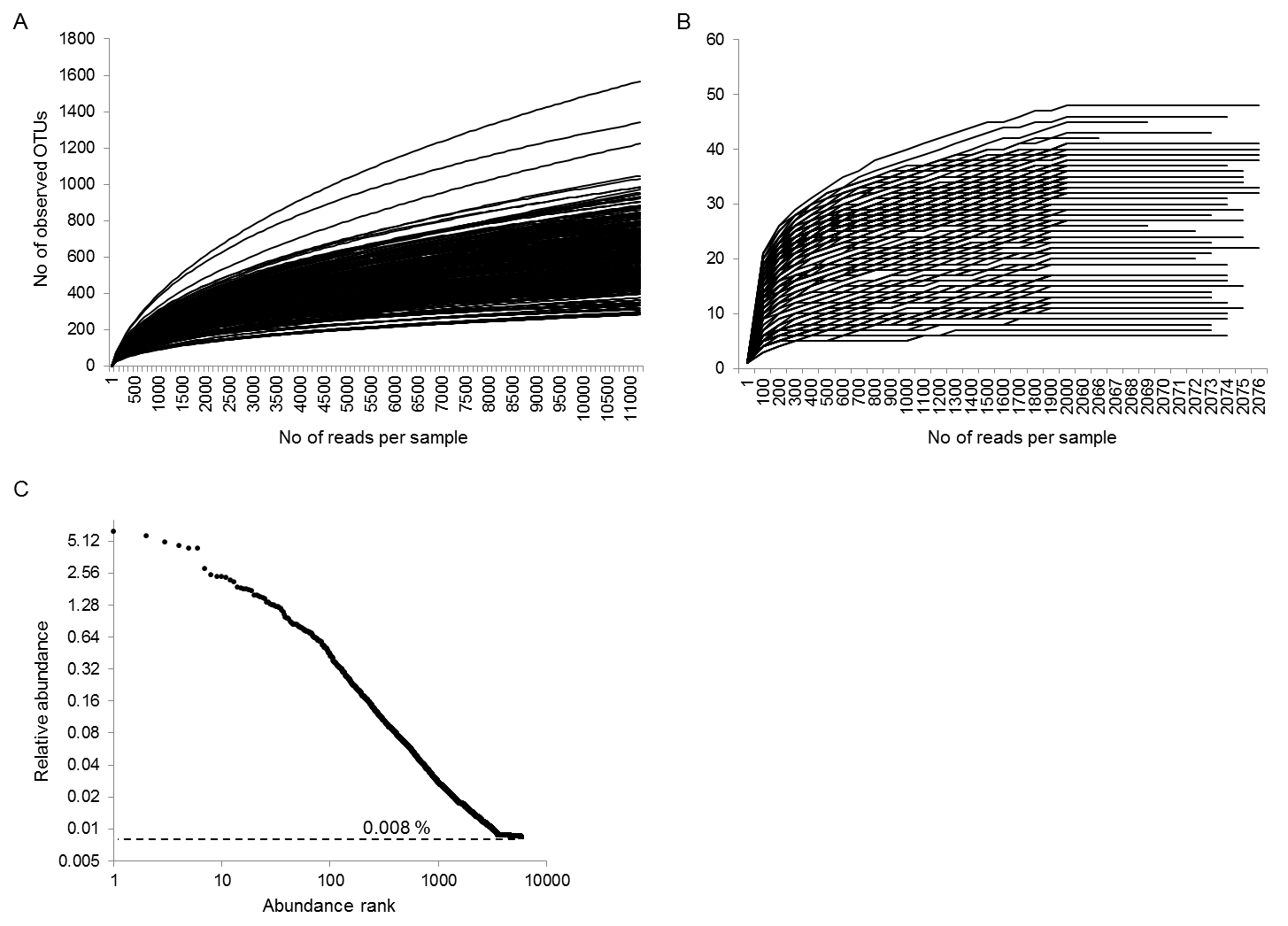
**

**Figure S6**

Rarefaction curves based on the calculated species richness (Sobs) for bacteria (A) and archaea (B). Rank abundance curve of bacterial OTUs (C).

**
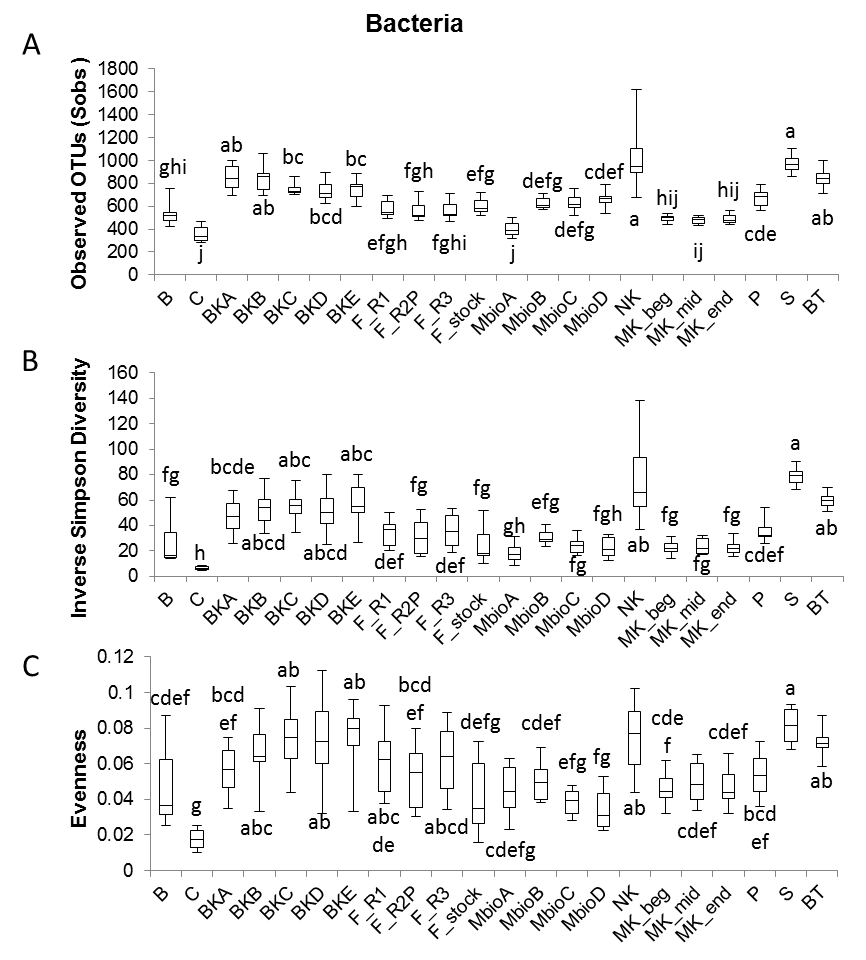
**

**Figure S7**

Median bacterial richness (A, Sobs), diversity (B, invsimpson) and evenness (C, invsimpsoneven) per reactor during one year monitoring survey. Statistical analyses were performed using Kruskal-Wallis test; boxplots holding the same label within a single panel do not differ significantly (p ≤ 0.05).

**
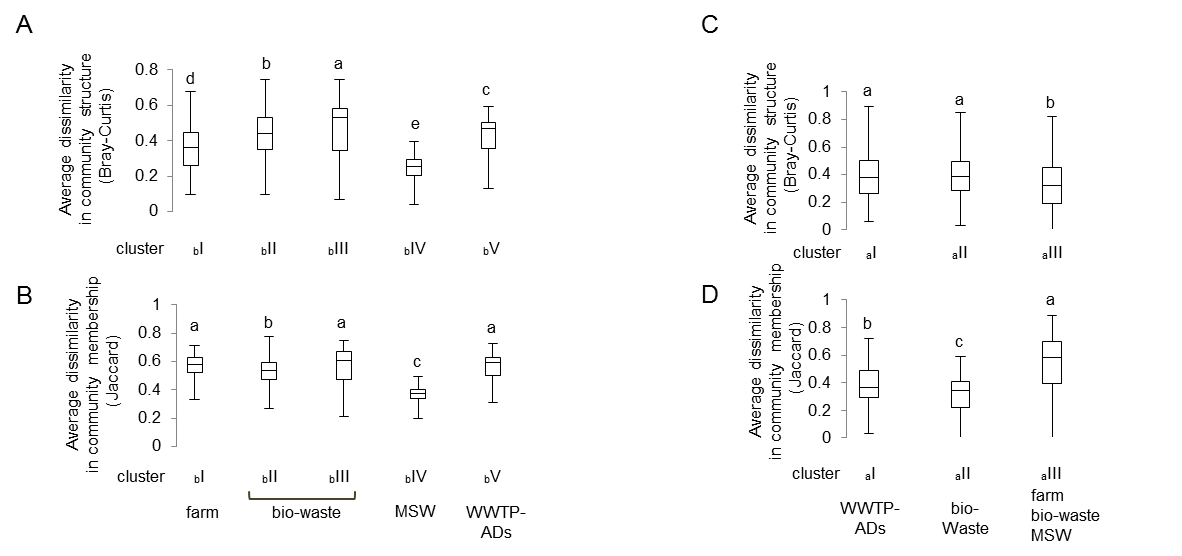
**

**Figure S8**

Cluster-specific average Bray-Curtis dissimilarity in community structures and Jaccard dissimilarity in community membership for bacteria (A, B) and archaea (C, D) for each of the community clusters. Statistical analyses were performed using Kruskal-Wallis test; boxplots holding the same labels within a single panel do not differ significantly (p ≤ 0.05).

**
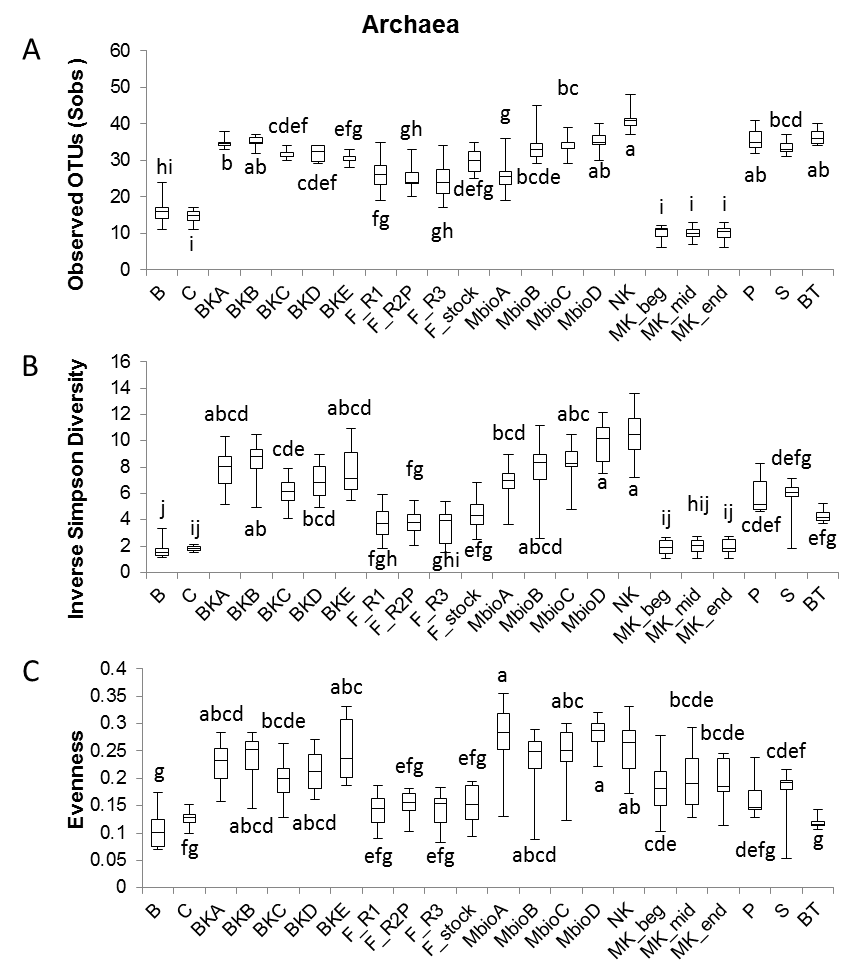
**

**Figure** **S9**

Median archaeal richness (A, Sobs), diversity (B, invsimpson) and evenness (C, invsimpsoneven) per reactor during one year monitoring survey. Statistical analyses were performed using Kruskal-Wallis test; boxplots holding the same labels within a single panel do not differ significantly (p ≤ 0.05).

**
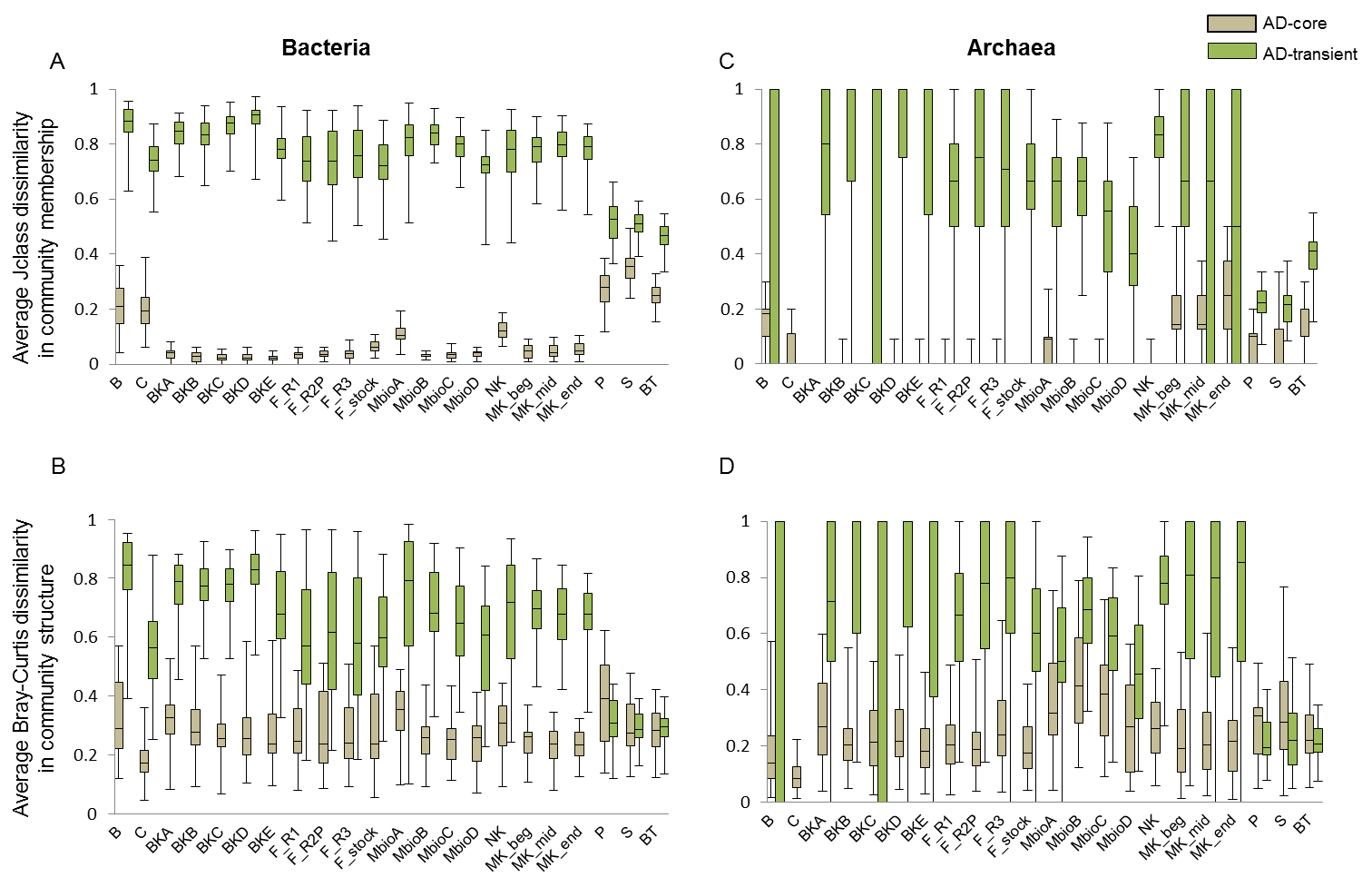
**

**Figure** **S10**

Calculated average Bray-Curtis dissimilarity in AD-core and AD-transient community structures and Jaccard (Jclass) dissimilarity in AD-core community and AD-transient membership for bacteria (A, B) and archaea (C, D) for each of the studied reactors.
